# Factors influencing clinical breast cancer screening: A cross-sectional study among Islamic women in Kumasi Metropolis of Ghana

**DOI:** 10.1371/journal.pone.0320726

**Published:** 2025-05-23

**Authors:** Abdul-Karim Abubakari, Janet Gross, Emmanuel Adusei-Poku

**Affiliations:** 1 Kwame Nkrumah University of Science and Technology, Kumasi, Ghana; 2 Morehead State University, Morehead, Kentucky, United States of America; Queensland University of Technology, AUSTRALIA

## Abstract

Clinical breast cancer screening among Ghanaian women is generally unsatisfactory due to poor knowledge despite its critical role in the early detection of breast cancer. Available studies in Ghana show that Islamic women have poorer screening rates which may be due to sociocultural and religious barriers. Understanding the factors that influence clinical breast cancer screening among Islamic women is a critical step that can help the design of initiatives to increase screening among Muslim populations in Ghana. Therefore, this study aimed to explore the factors influencing clinical breast cancer screening among Islamic women in the Kumasi Metropolis of Ghana. From August 20, 2024, to November 01, 2024, a community-based cross-sectional systematic sampling technique was deployed in the Aboabo and Asawase communities of the Ashanti Region to select 500 Islamic women for the study. Binary logistic regression was employed to determine the relationships between variables. Outcome variables with P-values < 0.05 were considered statistically significant. Most of the respondents were of Ghanaian Northern ancestry, with secondary-level education as the highest educational attainment. Compared to women with low cultural and religious norms, women with stronger personal and religious norms had 0.61 lower odds of screening (aOR=0.61, 95% CI = 0.34–1.08). Participant’s level of religiosity had a significant association with clinical breast cancer screening, with 1.16 times higher odds of screening (aOR= 1.16, 95% CI = 1.02–1.32) after adjusting for the covariates. Islamic women perceived high benefits of clinical breast cancer screening but fear of personal and social norm violations at the screening centers and poor knowledge about breast cancer limited their actual participation in clinical breast cancer screening practices. Implementing a national breast cancer education campaign to emphasize the need for asymptomatic or routine screening and provider training on culturally competent practices is encouraged.

## 1 Introduction

Women of African ancestry are more likely to be diagnosed with biologically aggressive phenotypes of breast cancer called triple-negative breast cancer [1]. Moreover, there is reason to believe that African ancestry itself may be linked to a hereditary susceptibility for particular patterns of breast cancer given the younger age distribution for the disease [2]. Concerns have been raised in Ghana about the increasing rates of breast cancer diagnoses coupled with an overburdened healthcare system that lacks multidisciplinary treatment programs or early detection, widening the mortality-to-incidence ratios in Ghana compared to other countries [1]. Life expectancy has increased as a result of Ghana’s demographic transition, which is marked by a change from high to low birth and death rates. Given that the risk of breast cancer (BC) rises with age, a longer life expectancy and better health outcomes are linked to a higher incidence of BC. Furthermore, a higher risk of BC has been associated with the intake of high-fat diets associated with modern lifestyles [1]. Other lifestyles associated with modernity that increase Ghanaian women’s risk of BC are the acceptance of a poorer lifestyle influenced by the Western way of living and increased adoption of reproductive patterns more common in Western populations such as delayed childbearing and decreased overall parity, which can increase BC risk in both pre-and postmenopausal women and has been associated with the increasing BC diagnosis in Ghana [1].

It should be highlighted that, while mammography is the recommended gold standard screening method, the cost is prohibitive and a barrier to clinical breast cancer screening (CBCS) in resource-limited settings such as sub-Saharan Africa [3,4]. As a result, the World Health Organization (WHO) [4] recommends CBCS and breast self-examination for women in resource-constrained environments. For the purposes of this study, CBCS refers to a breast examination performed by a healthcare expert to detect abnormalities. CBCS includes a visual inspection of skin and tissue and a medical expert’s manual check for atypical lumps and breast texture [5]. In Ghana, one study found just a 4.5% prevalence of CBCS among older women aged 50 and over [6]. The traditional African concept of the ‘ complete ‘ and attractive body is closely linked with the delayed presentation of BC diagnosis and screening in Ghana [7]. Perceptions of attractiveness and the ideal body shape vary among diverse cultures, reflecting specific social and cultural values [8]. Within Ghana’s conservative sociocultural context where a woman’s value is tied to her feminine features, losing either one or both breasts could have long-term consequences for social relationships as well as marriage [7]. Thus, the perceptions by men of diminished sexuality and femininity post breast cancer diagnosis may have been reasons for women’s unsatisfactory breast screening behaviors [7,9]. Unfortunately, mastectomy which is considered a mutilation of a woman’s body is the most common kind of BC therapy in sub-Sahara Africa [10]. Women also continue to underestimate their risk of breast cancer which hinders favorable attitudes toward early BC screening [10].

The significant societal stigma and cost to women’s relationships from a BC diagnosis in Ghana is one of the reasons women present at health facilities with advanced stages of BC with an average delay period of a year [11]. According to Dzidzornu et al [12], Ghanaian women are highly aware of BC [12]. Awareness campaigns are annually organized in October, which is declared BC awareness month. According to Boamah Mensah et al [13] and Dzidzornu et al[12], the Breast Cancer Month awareness campaigns focus primarily on screening instead of holistic education on BC including the benefits of asymptomatic screening. Meanwhile, there is low mammography awareness resulting from Ghana’s limited nationwide mammography services [14]. Due to the limited mammography awareness [12] only 2% of Ghanaian women self-report to health facilities for mammography screenings [15]. The burden of BC in Ghana (incidence 43% vs mortality 17.7 cases per 100,00 women) relative to the global incidence and mortality (11.6% vs 6.6%) is greater as the disease is often not detected until an advanced stage [16].

Islamic women have had limited decision-making power within their families [17–19]. Islam is viewed as a supporter of patriarchy and has created gender roles and obligations that have endured throughout the years limiting the expression of women [20,21]. A comparative analysis of women’s educational attainments between Muslims and Christians in the West Africa sub-region has shown that Islamic women received less formal education compared to their Christian counterparts [22,23]. An education disparity between Muslims and Christians has persisted over time [22,23]. Between 2005 and 2016, Muslims in the West African sub-region experienced a decline in educational attainment compared to Christians, with Muslims receiving less than six years of formal education on average [23]. This discrepancy in educational attainment has been sustained due to child marriage, poverty from a lack of formal employment opportunities, limited resources for raising a large family, and prioritization of male child education over that of the girl child [[23,24]]. Cultural attitudes prevalent in Ghana that prevent Muslim girls from pursuing higher education include negative self-perceptions that are reinforced in schools, inaccurate expectations placed on Muslim girls’ abilities by teachers, and expectations of Muslim girls’ roles in the community and nation [25–27]. Families perceived the formal education of adolescent girls to be un-Islamic and a waste of limited resources [28]. The acceptance of child marriage in Islamic families with the primary objective of preventing premarital sex and pregnancy worsens women’s educational attainment and gender equality gaps [23,29]. Early marriage is encouraged in Islam for their honor to the family legacy and economic benefits, whereas single status or late marriage is associated with shame for families of women [29]. Girls marrying as minors are at significantly higher risk for unfavorable health outcomes due to early pregnancy coupled with a lack of employable skills limiting their access to healthcare [30,31]. Results of a case-control study showed that women with breast cancer had histories of early marriages with associated early sexual exposure (at under 18 years of age), compared to the control group [32]. Furthermore, child marriage was one of the most common extrinsic factors in a case-control study that modulated breast cancer risk with an increasing odds of 2.5 [33].

Data is scarce about the prevalence of breast cancer and screening rates among Islamic women in the Kumasi Metropolis. The difference between Islamic women’s interpretation of Quranic texts and their health-seeking behavior is a complex interplay of fear of cancer diagnosis, knowledge, health-seeking care behavior, and adherence to religious codes on modesty [9,34]. While Islamic teaching reinforces principles that advocate for health-seeking, sociocultural influences such as cultural taboos, myths, and misconceptions about BC can influence behaviors that diverge from these guidelines [9]. Regardless of the Qur’an or other relevant texts, Ghana’s diverse Islamic populations may identify with Islam based on their realities. In an earlier study, religion was associated with respondents’ lower perceived risk of breast cancer and the benefits of BC screening uptake in the Volta region of Ghana [35]. Moreso, a striking disparity was observed in the utilization of BC screening services between Muslim women and their Christian counterparts in the Kumasi Metropolis of the Ashanti region [36]. The outcome of the work of Gyedu et al [14,36] highlights the impact of various sociocultural factors including the Islamic religion on BC screening [36]. Nevertheless, Gyedu et al’s [14,36] works were silent on the screening disparity between Islamic women and Christians [36]. It is envisaged that the sociocultural approach of this study would contribute to the limited scientific and context-specific evidence on the socio-religious challenges Islamic women face with BC screening in Ghana and provide evidence on best practices that could address the practical barriers to BC screening in the Asokore Mampong Municipality. It is against this backdrop that this study was undertaken to understand the factors that influence clinical breast cancer screening among Islamic women in the Kumasi Metropolis of Ghana.

## 2 Materials and methods

### 2.1 Ethical considerations

This study was ethically approved by the Committee on Human Research Publication and Ethics at the School of Medicine and Dentistry, Kwame Nkrumah University of Science and Technology (CHRPE/AP/1329/24). The study was conducted in conformity with the declaration of Helsinki. Permissions were obtained from the Municipal Health Director of the Asokore Mampong Municipal Assembly, Kumasi. Participants were provided with information on the purpose and nature of the study. Each participant signed an informed consent form before data were collected. The anonymity of respondents and confidentiality were ensured by ensuring that personal information was kept safe on a computer secured with a password changed every 3 days. The principal investigator was the only researcher with access to respondents’ personal information.

### 2.2 Theoretical model

**Fig 1:**
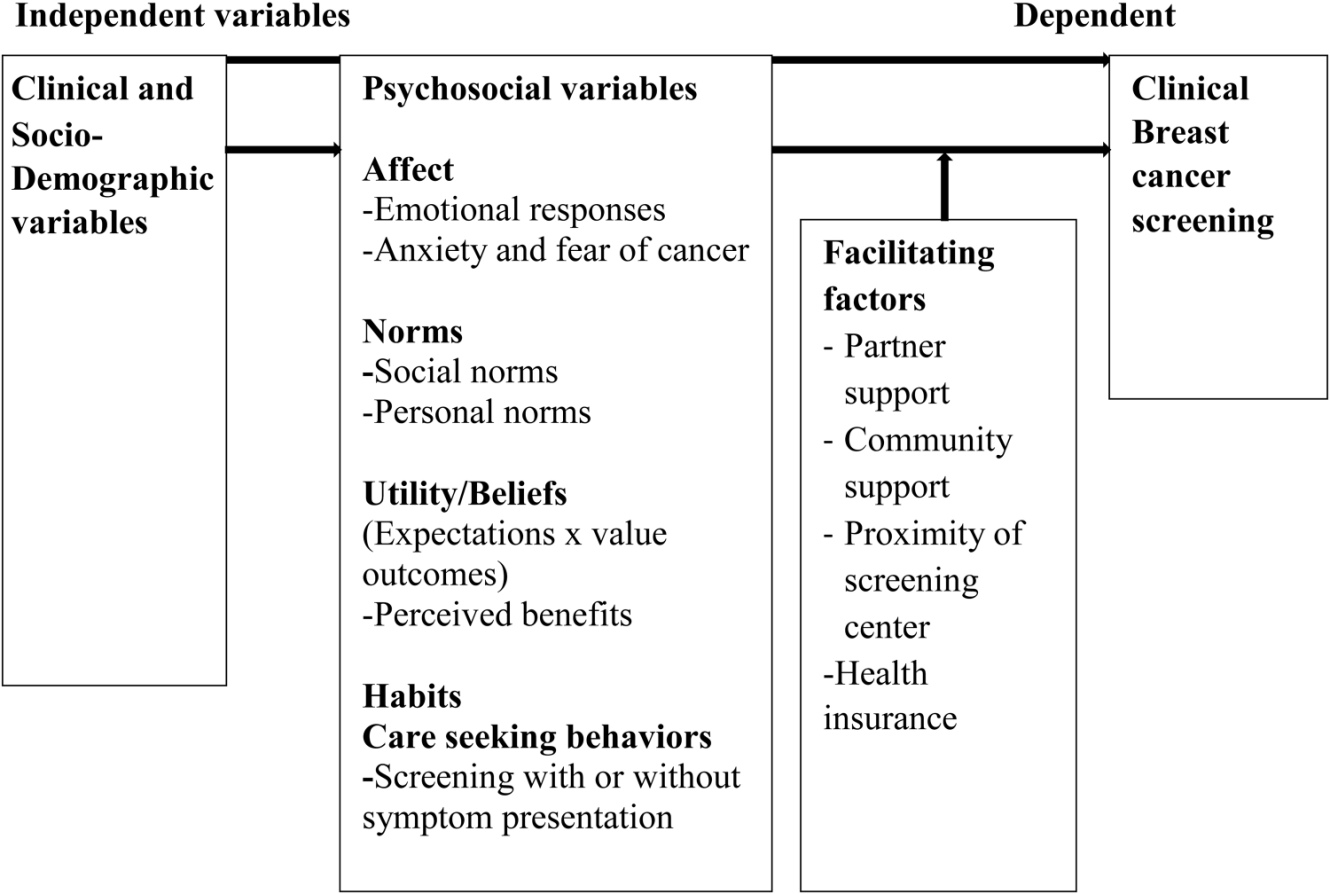
The conceptual framework adopted from the Theory of Care Seeking Behavior (TCSB) [37].

Based on Triandis’ Theory of Interpersonal Behavior, Lauver developed the Theory of Care Seeking Behavior (TCSB)[37] to describe care-seeking behavior in relation to clinical and demographic characteristics, external conditions, and psychosocial concepts. The TCSB is predicated on the idea that people will participate in preventive behavior if they: (1) generally practice healthy habits; (2) feel confident about the behavior’s outcome and have no negative emotions related to performing it; (3) feel supported by important others in doing so; and (4) have favorable sociodemographic circumstances. Additionally, population behavior on screening tests has been explained by the TCSB [38]. The Theory of Care Seeking Behavior includes four main constructs that suggest that psychosocial variables such as affect, utility (expectations x value outcomes), norms, and habits can predict care-seeking behavior (Fig. 1). In other words, the psychosocial factors can interact with facilitating factors to predict care-seeking behavior. Clinical and sociodemographic characteristics, such as age, ethnicity, and economic status, do not predict care-seeking because any influences of clinical and sociodemographic factors are captured by the salient psychosocial and facilitating factors more proximal to care-seeking [37]. Psychosocial and facilitating factors are more readily modifiable than clinical and sociodemographic variables by educational programs designed to increase the number of women who seek cancer screening [37].

Since CBCS activity is a secondary preventive behavior as opposed to a circumstance involving illness, this study used TCSB to describe and predict CBCS behavior. The primary TCSB constructs—habits, affect, norms, and utility (perceived benefit) —served as the study’s conceptual framework constructs. The conceptual framework used to guide this study is shown in Fig 1 and is based on a modification of the TCSB. The theory of TCSB was chosen because data suggests that it can more successfully address research concerns, particularly in environments with limited resources.

The constructs have been operationally defined as follows. Affect relates to the feelings that an Islamic woman experiences when seeking care, such as anxiety over a possible positive diagnosis or embarrassment about an examination. Utility indicates the sum of corresponding expectations and values around BC care-seeking outcomes for Islamic women, and value refers to the significance of those outcomes. For this study, the perceived benefit construct describes a person’s conviction that specific favorable outcomes would occur from a particular behavior. The term perceived benefit was used in place of the term utility to enhance understanding of the utility construct of TCSB. The perceived benefit of CBCS is closely related to expectation and value outcomes because it describes the positive outcomes that an individual expects to obtain from seeking care (e.g., screening). In other words, the perceived benefit construct/ beliefs pertain to the potential outcomes of seeking care or participating in health behaviors, such as the advantages of early detection, treatment, or improved health. Normative influences include both social and personal norms, as well as interpersonal agreements to seek care. Social norms are other people’s beliefs about care-seeking. Personal norms are one’s personal opinions about what is morally proper when seeking care. Habit relates to how an individual acts when experiencing symptoms (for example, whether or not one gets medical attention right away). Habit reflects one’s regular care-seeking conduct as well as previous care-seeking experiences. Facilitating factors are precise, objective, external conditions that allow one to seek care, such as proximity to screening facilities, without which care-seeking will be hampered.

Clinical factors include current and prior conditions such as family history of cancer, and personal history of abnormal findings from screening that have been linked directly to cancer screening [37,39]. However, clinical factors in the original model were replaced with participants’ prior knowledge about breast cancer, reproductive, lifestyle, and health services-related factors because environmental, reproductive, hormonal, and lifestyle factors have all been linked to individual chances of breast cancer [40]. Furthermore, Lauver argued that cancer screening behavior could be explained solely by variables external to the theory through mediation with the four main theoretical variables [37]. Knowledge (inversely associated with breast cancer risk) as a composite variable, reproductive, lifestyle, and health services-related factors combined with sociodemographic data from Islamic women, is likely to improve understanding of Islamic women’s CBCS behaviors.

### 2.3 Study area

From August 20, 2024, to November 01, 2024, a cross-sectional approach was used to select Islamic women in two settlements in the Asokore Mampong Municipality of the Ashanti Region. Indigenous religious beliefs, whereas the majority of people in the north identify as Muslims [41]. The Ashanti Region of Ghana is the country’s second-largest and one of the most Islamically diverse regions, with a population of 5,440,463 [42]. People of Ashanti ethnicity dominate the region and account for 39.3% of Ghana’s total Akan population. According to GhanaWeb [43], Kumasi’s ethnic and cultural variety is influenced by the city’s strategic location and expanding urbanization, as well as the capacity of numerous ethnic groups to coexist and share cultural values. This study was conducted in the Asokore Mampong Municipality, one of the Ashanti Region’s forty-three municipalities, with Kumasi as its capital. The Asokore Mampong Municipality accounts for approximately 11.2% of the region’s culturally diverse population [[42]. The municipality is made up of fourteen clusters of communities with an estimated population of 42,037 people, the majority of whom are Akan (40.9%), followed by people from Northern Ghana (36.7%), Guans (10.7%), Ewes (3.0%), and Ga Dangme (4.6%). The Islamic faith has the highest representation in the municipality, with 55.4% of residents, followed by Christians at 41.8%, and other religious groupings at 2.8% [42].

### 2.4 Study population

This study was conducted in the Asokore Mampong Municipality, one of the Ashanti Region’s forty-three districts, with Kumasi as its capital. The Asokore Mampong Municipality accounts for approximately 11.2% of the region’s culturally diverse population [42]. The Islamic faith has the highest representation in the municipality, with 55.4% of residents.

### 2.5 Inclusion criteria

a. Practicing Islamic women aged 18 years and above.
b. Islamic women from other parts of Africa who can speak either Twi, Hausa, or English.
c. Ghanaian Islamic women who can speak either Twi, Hausa, or English.
d. Residents in the Asawase, and Aboabo communities for at least 12 months
e. Respondents with the above characteristics willing and consented to participate in the study were enrolled.
f. Women without any form of visual or hearing impairments

### 2.6 Sample size determination

Data from the Ghana Statistical Service, 2020 Population and Housing Census, showed that 544,728 (55.4%) of the population in the Asokore Mampong Municipality are Muslims aged 18 years and above [42]. The sample size was estimated using Slovin’s mathematical formula to determine the minimum sample size n = N/ (1 + Ne2), where n is the sample size, N (544,728) is the population size, and e (0.05) is the level of precision. The estimated sample size was then 544,728/1 + 544,728 (0.05)^2^ = 399.07. Assuming a non-response rate of 10%. An adjustment for non-response = 399.07/0.9. Thus, the estimated sample size was = 445. The sample size was overestimated to 500 to allow for greater diversity within the sample and non-response rate, which improves the generalizability of the findings to a broader population.

### 2.7 Data collection tool and sampling

A two-stage selection approach was employed to first identify communities and second to identify respondents from each of the communities. During stage one, two communities were chosen out of six communities home to Islamic populations through balloting. The principal investigator with two field officers who were experts in both Hausa and three major languages spoken in both communities sampled respondents from both Asawase and Aboabo.

In stage two, cross-sectional simple random and systematic proportional sampling techniques were deployed to select participants from each selected household in the two communities. The household sampling intervals for both study sites were determined by the quotient of the number of households (N) and divided by sample size (n). Every N/n ^th^ household was selected for the study. For instance, for the Aboabo community, the sampling interval was determined as follows: 6,626/500 = 13^th^ households, while for Asawase, the sampling interval was determined as 9144/ 500 = 18^th^ households. The first household to be visited at Aboabo was determined by a random method. A number between 1–10 was assigned to the respondents and the respondent selected the first house based on counting that number from the closest house to the drop-off point. The population sizes for eligible women between the ages of 18 and above years at Asawase and Aboabo were 46, 315, and 34, 206 respectively, with a total population of the two communities as 80, 521. The proportionate number of respondents to be recruited from each migrant community was determined as shown below:


$$Asawase=\frac{46315\times 500}{80,521}\approx 288$$

$$Aboabo=\frac{34206\times 500}{80,521}\approx 212$$

Any Islamic woman who met the inclusion criteria from each of the 15th and 20th households in Aboabo and Asawase respectively and expressed interest in the study was enrolled. This process was repeated separately for each of the two communities until the required sample size for each of the communities was attained. First, for each household, the list of eligible women was retrieved from the head of the household. The respondents to be selected from each household were determined using probability sampling proportional to the total number of eligible respondents for that community. The number of respondents for each community was obtained by dividing the number of eligible women in each community by the total number of eligible women in the two selected communities and multiplying by the estimated sample size. To ensure that each eligible respondent had an equal chance of being selected, unique identifiers were assigned to all the names of respondents in each household. The unique identifiers were put in a bowl and randomly drawn. Respondents whose unique identifiers were drawn and met the inclusion criteria were invited to participate. Eligible women varied in age, profession, and different religious sects including different degrees of adherence to their cultural and religious values. A written consent was provided, and participation was entirely voluntary.

The principal investigator or a member of the research team personally invited women to participate in the recruitment process through the community address system. The heads of households and the employees of the Asokore Mampong Municipal Assembly assisted the process by sending out study leaflets developed by the research team informing eligible women that a researcher would be visiting their homes and encouraging them to volunteer. In addition, heads of households provided physical areas where the questionnaire could be completed. The respondents who agreed to participate in the study were enrolled. Each questionnaire had a 15-minute completion time. The process was repeated until the proportionate estimated sample size for each migrant community relative to the total sample size was obtained. The questionnaires were completed during a time that did not interfere with family or household activities. All 500 questionnaires issued were completely and accurately filled out and returned on the spot. As a result, a 100% response rate was reached. Data collection began in Asawaase, followed by that of Aboabo, and lasted around eight weeks. The researchers felt that this sampling procedure was the most effective for fulfilling the study objectives since it was likely to yield an objective outcome that was representative of the target population and could be generalized. Furthermore, the sample technique deployed offered respondents equal opportunity to be selected to participate in the study.

A modified version of the validated self-assessment tool for cervical cancer risk originally developed by Purwandari et al [44] and adopted by several researchers were used as the data collection tool. The resulting questionnaire consisted of three sections. The first section consisted of items exploring participants’ demographic and background characteristics including age, level of religiosity, education, religious sect, occupation, and ethnic affiliation. The second section explored participants’ reproductive, lifestyle-related, and behavioral-related factors. Questions on reproductive health history, lifestyle choices, and behavioral-related factors were dichotomous variables (Yes/No). Each item had a response option of “No” and “Yes”. Responses were scored as either correct (1) or wrong (0) based on facts identified from the literature. A composite score was determined by summing the number of correct responses against the total number of correct responses. A correct response was determined by correctly identifying facts about BC. The third section based on earlier work by Dsouza et al[45] explored participants’ knowledge and risk factors for breast cancer (awareness, cause, risk factors, and clinical features). Respondents were asked to choose the best level of agreement with each statement from a 7-point Likert scale. For positive statements, strongly agree = 7, agree = 6, slightly agree = 5, neutral = 4 slightly disagree = 3, disagree = 2, and strongly disagree = 7. The reverse score was used for negative statements. Based on options respondents believed to be their correct options and compared with correct answers based on the literature, a correct response was categorized as low risk, while an incorrect response was categorized as high risk. The tool consisted of questions that explored participants’ core knowledge about breast cancer and breast screening behavior. To validate the resulting questionnaire, which included the respondents’ sociodemographic characteristics and the main constructs of the TCSB among ethnically varied women, the study team translated and culturally adjusted the research instruments. The instrument usability, content validity, and conceptual construction validity were all evaluated through instrument piloting. The original tool was translated from English to Hausa and Twi (predominant languages spoken in the study communities) by the research team and health sciences language expert from the Kwame Nkrumah University of Science and Technology, who also did an independent back-translation to English to ensure accuracy. An expert group made up of Hausa and Twi language translators from the Ghana Institute of Languages and the Kwame Nkrumah University of Science and Technology reached a consensus and blended the two forward-translated versions to create the Twi and Hausa versions. This was done to create the best possible instrument. A multidisciplinary four-member committee in Ghana, including experts in transcultural psychiatry and family medicine, examined the questionnaire during the translation, reverse translation, and overall questionnaire assessment procedure. To examine for potential mistranslations and language misunderstandings, the research team observed the procedure and deliberated on the question items and data during regularly planned meeting sessions. After validation, the questionnaire was distributed in the two research locations from January to March 2024. The Cronbach’s Alpha was used to determine the reliability of the scales. The Cronbach’s Alpha values for perceived benefits, knowledge, habits, norms, facilitating factors, and affect were 0.788, 0.694, 0.813, 0.810, 0.828, and 0.817 respectively indicating adequate reliability.

### 2.8 Data quality control

The research questionnaire was meticulously designed and pretested to ensure data quality. Before beginning fieldwork, the lead investigator provided two assistant field officers with ten days of rigorous training on the study’s goal, data collection instrument, sampling techniques, and suitable methods of eliciting responses from study participants. The questionnaire was pretested on 10% of the total sample size at another similar community in the Ejisu-Juabeng Municipality of the Ashanti Region to ensure that the data collection tool assessed the necessary information for the study and that any ambiguous language was addressed. There were no significant revisions to the original draft.

### 2.9 Data analysis and presentation

Descriptive analysis such as frequencies and percentages were used to analyze the sociodemographic characteristics and the main constructs of the TCSB. A further analysis establishing an association between respondents’ sociodemographic factors, and reproductive and behavioral factors was carried out. Data were analyzed using SPSS version 27. Continuous data were summarized using mean, median, and standard deviation, and categorical data were summarized with frequencies and percentages. Reliability analysis was deduced by computing Cronbach Alpha (α) and composite reliability. Scales with Cronbach α and composite reliability of 0.8 and 0.70 respectively are considered to have met the minimum threshold [46]. In this study, the Cronbach α and composite reliability of the construct items mostly exceeded 0.8 indicating that the scale had satisfactory reliability. To rank knowledge, perceived benefits, norms, affect, facilitating factors, and habits, each construct was assigned an overall median score with which each item’s responses were compared. A score below the overall median for that construct was categorized as low, while a score above or equal to the overall median was considered high. For instance, for the knowledge construct, respondents’ responses to the knowledge items were weighted and scored for each response. A correctly answered question was awarded a weighted score. The scores were subsequently summed to generate the total score from which the composite median was deduced. Respondents’ total scores were then compared with the composite median for knowledge to determine knowledge ranking. Respondents with scores below or equal to the median were labeled as low BC knowledge while those with scores above the median were labeled as high BC knowledge. To estimate the predictors of knowledge, an assumption is that respondents’ interface with healthcare providers could increase their knowledge of BC. Based on the literature, selecting variables pertaining to respondents’ reproductive, behavioral, and health services-related factors was made and tested. The Chi-square and Mann-Whitney U tests of association were initially carried out. Variables with multicollinearity were eliminated. However, variables that were significant at p-value 0.05 in the model were further regressed unto a binary logistic regression to estimate the independent and combined effects of the predictors of knowledge. Additionally, predictors of clinical breast screening were determined relative to the main TCSB constructs for the study population using the Chi-square test and Mann-Whitney U test. This was achieved by scoring the initial responses from the main TCSB domains and splitting at the median. The relationship between the outcome and independent variables established by a p-value <0.05 indicated the presence or absence of a domain. A binary logistic regression was further carried out to explore the relationship and the strength of the variables associated with women’s willingness to undergo CBCS. Variables that were significant at p < 0.05 in the logistic model were regressed on CBCS predictors to determine the association’s strength.

## 3 Results

### 3.1 Socio-demographic Information and Association with Clinical Breast Cancer Screening

Five hundred practicing Islamic women aged 18 years and above residents in Asokore Mampong Municipality of Kumasi were sampled for the study. Table 1 shows the association between clinical breast cancer screening and the socio-demographics of the study participants. A significant majority have never been screened. Most of the women were between the ages of 18 and 27 years with the most earning (30.4%) less than GH¢366 monthly. Income was statistically significant with CBCS (p-value = 0.020). More than half of the participants were single. Almost half of the women were affiliated with the Ahlus Sunna Wal-Jama’a sect of Islam (45.2%). Using a numeric scale of 1–10, and median scores of 9 and 10 for the level of religiosity and the role that Islam played in respondents’ lives respectively indicated that participants were extremely religious and that Islam significantly influenced their life decisions including health. Both the religiosity and the extent to which Islam informed their life decisions were significantly associated with CBCS. The majority of women (67%) were of Northern ancestry with high school certificates as their highest level of educational attainment. Moreover, more than one-fourth of the participants were self-employed and single (n = 256, 51.2%). The details are summarized in Table 1 below.

**Table 1:** Socio-demographic information and association with clinical breast cancer screening.

		Clinical Breast cancer screening		
	Total (500)	No (352)	Yes (148)	
Variables	N (%)	n (%)	n (%)	p-value
Age				0.343
18-27	298 (59.6)	201 (40.2)	97 (19.4)	
28-39	118 (23.6)	86 (17.2)	32 (6.4)	
40-50	52 (10.4)	41 (8.2)	11 (2.2)	
51-61	24 (4.8)	17 (3.4)	7 (1.4)	
> 61	8 (1.6)	7 (1.4)	1 (0.2)	
Income				**0.020**
less than GH¢366	152 (30.4)	95 (19.0)	57 (11.4)	
Between GH¢366–500	113 (22.6)	87 (17.4)	26 (5.2)	
Between GH¢ 501–700	69 (13.8)	56 (11.2)	13 (2.6)	
Between GH¢701–900	55(11.0)	40 (8.0)	15 (3.0)	
GH¢905 and above	111(22.2)	74 (14.8)	37 (7.4)	
Marital Status				**0.017**
Married	200 (40.0)	151 (30.2)	49 (9.8)	
Single	259 (51.8)	166 (33.2)	93 (18.6)	
Cohabiting	9 (1.8)	8 (1.6)	1 (0.2)	
Widowed	22 (4.4)	19 (3.8)	3 (0.6)	
Divorced	10(2.0)	8 (1.6)	2 (0.4)	
Polygamous marriage^¥^				0.141
Yes	84 (36.2)	69 (29.7)	15 (6.5)	
No	148 (63.8)	109 (47.0)	39 (16.8)	
Sect of Islam				**0.033**
Orthodox	102 (20.4)	79 (15.8)	23 (4.6)	
Ahlus Sunna Wal-Jama’a	226 (45.2)	152 (30.4)	74 (14.8)	
Tijaniyya	40 (8)	31 (6.2)	9 (1.8)	
Ahmadiyya	121 (24.2)	80 (16.0)	41 (8.2)	
Shia	10 (2)	10 (2.0)	0 (0.0)	
Other	1 (0.2)	0 (0.0)	1 (0.2)	
Level of religiosity ((median (Q1, Q3)) *	9 (6, 10)	8 (6, 10)	10 (8, 10)	**<0.001**
Role of religion level ((median (IQR)) *	10 (7, 10)	10 (7, 10)	10 (9, 10)	<**0.001**
Employment status				**0.039**
Self-employed	177 (35.4)	138 (27.6)	39 (7.8)	
Unemployed	18 (3.6)	11 (2.2)	7 (1.4)	
Government employee	93 (18.6)	57 (11.4)	36 (7.2)	
Private sector employee	25 (5.0)	15 (3.0)	10 (2.0)	
Student	175 (35)	121 (24.2)	54 (10.8)	
Housewife	12 (2.4)	10 (2.0)	2 (0.4)	
Occupation				<**0.001**
Petty trading	104 (20.8)	87 (17.4)	17 (3.4)	
Fishmonger	5 (1.0)	5 (1.0)	0 (0.0)	
Dressmaking	49 (9.8)	36 (7.2)	13 (2.6)	
Farmer	7 (1.4)	5 (1.0)	2 (0.4)	
Artisan	14 (2.8)	4 (0.8)	10 (2.0)	
Professional	101 (20.2)	50 (13.6)	51 (10.2)	
Hairdressing	22 (4.4)	17 (3.4)	5 (1.0)	
Participants with no occupation	198 (39.6)	148 (26.0)	50 (10.0)	
Level of Education				**<0.001**
No formal education	19 (3.8)	15 (3.0)	4 (0.8)	
Primary education	40 (8.0)	37 (7.4)	3 (0.6)	
Junior high school/middle school	54 (10.8)	43 (8.6)	11 (2.2)	
High school, but did not graduate	22 (4.4)	19 (3.8)	3 (0.6)	
High school graduate	161 (32.2)	122 (24.4)	39 (7.8)	
HND/Diploma or 3-year degree	75 (15.0)	43 (8.6)	32 (6.4)	
4-year college graduate	105 (21.0)	60 (12.0)	45 (9.0)	
More than a 4-year college degree	24 (4.8)	13 (2.6)	11 (2.2)	
Ethnic Affiliation				**0.001**
Northern descent	335 (67)	251 (50.2)	84 (16.8)	
Ashanti	71 (14.2)	34 (6.8)	37 (7.4)	
Akan ancestry other than Ashanti	39 (7.8)	28 (5.6)	11 (2.2)	
Ga	7 (1.4)	4 (0.8)	3 (0.6)	
Ewe	11 (2.2)	9 (1.8)	2 (0.4)	
Non-Ghanaian	31 (6.2)	22 (4.4)	9 (1.8)	
Other Ghanaian ethnic groups such as Guan, Nafana, Avatime, Efutu	6 (1.2)	4 (0.8)	2 (0.4)	

* Median (Q1, Q3); Q1 = 1st quartile, Q3 = 3rd quartile, and p-value obtained using Mann-Whitney U test with significance level (α = 0.05). All other values were presented in frequency and percentages with p-values obtained using the chi-square test (α = 0.05).

### 3.2 Reproductive, Lifestyle, and Behavioral-related Factors

Table 2 presents the reproductive, lifestyle, and behavioral-related factors. A significant majority of the women had neither had an HIV test nor a history of current or past contraceptive use. The majority of neither smoked nor consumed alcohol with more than three-quarters of them being aware of breast cancer disease (87.4%). Almost half of the participants (41.6%) did not know whether there were any health services available for clinical breast cancer screening (CBCS). Age of first marriage and parity were statistically associated with CBCS (see Table 2 for details).

**Table 2:** Reproductive, lifestyle and behavioral-related factors.

		Clinical Breast Cancer Screening		
	Total (500)	No (352)	Yes (148)	
Variables	N (%)	n (%)	n (%)	p-value
Age of first marriage				**0.003**
Never been married	269 (53.8)	175 (35.0)	94 (18.8)	
< 20 years old	68 (13.6)	58 (11.6)	10 (2.0)	
≥20 years old	163 (32.6)	119 (23.8)	44 (8.8)	
Age of menarche (median (Q1, Q3)) *	14 (13, 15)	14 (13, 15)	14 (13, 15)	0.612
Have children				**0.001**
Yes	231 (46.2)	179 (35.8)	52 (10.4)	
No	269 (53.8)	173 (34.6)	96 (19.2)	
Age of first childbirth				0.420
14-20	71 (30.7)	46 (19.9)	25 (10.8)	
21-25	120 (52.0)	87 (37.7)	33 (14.3)	
≥ 26	40 (17.3)	30 (13.0)	10 (4.3)	
Number of children				0.167
1	39 (16.9)	29 (12.6)	10 (4.3)	
2	64 (27.7)	39 (16.9)	25 (10.8)	
3	45 (19.5)	27 (11.7)	18 (7.8)	
4 and above	83 (35.9)	44 (19.0)	39 (16.9)	
Smoking				0.630
Never	490 (98.0)	344 (68.8)	146 (29.2)	
No, but a former smoker	3 (0.6)	3 (0.6)	0 (0.0)	
Yes, occasionally at events	6 (1.2)	4 (0.8)	2 (0.4)	
Yes, daily	1 (0.2)	1 (0.2)	0 (0.0)	
Type of foods consumed				
Fast Foods				**0.042**
Yes	212 (42.4)	139 (27.8)	73 (14.6)	
No	288 (57.6)	213 (42.6)	75 (15.0)	
Fruits and vegetables				**0.003**
Yes	316 (63.2)	208 (41.6)	108 (21.6)	
No	184 (36.8)	144 (28.8)	40 (8.0)	
Local dishes				0.841
Yes	458 (91.6)	323 (64.6)	135 (27.0)	
No	42 (8.4)	29 (5.8)	13 (2.6)	
Mainly fast foods and canned foods				0.463
Yes	48 (9.6)	36 (7.2)	12 (2.4)	
No	452 (90.4)	316 (63.2)	136 (27.2)	
Frequency of fruits and vegetables consumption				**0.008**
Never	32 (6.4)	21 (4.2)	11 (2.2)	
Less than once per week	1 (0.2)	0 (0.0)	1 (0.2)	
1-2 times per week	186 (37.2)	130 (26.0)	56 (11.2)	
3-4 times per week	110 (22.0)	66 (13.2)	44 (8.8)	
5-6 times per week	24 (4.8)	19 (3.8)	5 (1.0)	
7 or more times per week	37 (7.4)	25 (5.0)	12 (2.4)	
I don’t know	110 (22.0)	91 (18.2)	19 (3.8)	
Alcohol consumption				0.337
Never	464 (92.8)	323 (64.6)	141 (28.2)	
No, but I used to drink	19 (3.8)	16 (3.2)	3 (0.6)	
Yes, occasionally at events	17 (3.4)	13 (2.6)	4 (0.8)	
Exercise				**0.049**
No, not at all	172 (34.4)	118 (23.6)	54 (10.8)	
Yes, I do at least 30 minutes of moderate-intensity aerobic activity 5 days a week	61 (12.2)	39 (7.8)	22 (4.4)	
Yes, I do at least 30 minutes of moderate-intensity aerobic activity less than 5 days a week	37 (7.4)	21 (4.2)	16 (3.2)	
Yes, I do brisk walking exercises occasionally	230 (46.0)	174 (34.8)	56 (11.2)	
Age of sexual debut				**0.004**
Never been sexually exposed	126 (25.2)	86 (17.2)	40 (8.0)	
Below 15	23 (4.6)	20 (4.0)	3 (0.6)	
15-20	242 (48.4)	183 (36.6)	59 (11.8)	
21-25	97 (19.4)	57 (11.4)	40 (8.0)	
26-30	12 (2.4)	6 (1.2)	6 (1.2)	
HIV tests				**0.031**
Never	290 (58.0)	219 (43.8)	71 (14.2)	
Yes, less than 3 months ago	19 (3.8)	10 (2.0)	9 (1.8)	
3 months ago,	9 (1.8)	5 (1.0)	4 (0.8)	
6 months ago,	12 (2.4)	8 (1.6)	4 (0.8)	
Yes, more than 6 months ago	24 (4.8)	12 (2.4)	12 (2.4)	
Yes, a year ago	22 (4.4)	13 (2.6)	9 (1.8)	
Yes, more than a year ago	124 (24.8)	85 (17.0)	39 (7.8)	
Current use of hormonal contraceptives				0.066
Yes	150 (30.0)	97 (19.4)	53 (10.6)	
No	350 (70.0)	255 (51.0)	95 (19.0)	
Past use of hormonal contraceptives				**0.007**
Yes	144 (28.8)	89 (17.8)	55 (11.0)	
No	356 (71.2)	263 (52.6)	93 (18.6)	
Lump in breast				0.541
Yes	32 (6.4)	21 (4.2)	11 (2.2)	
No	468 (93.6)	331 (66.2)	137 (27.4)	
Heard about breast cancer				**0.011**
Yes	437 (87.4)	299 (59.8)	138 (27.6)	
No	63 (12.6)	53 (10.6)	10 (2.0)	
Heard about mammography				**<0.001**
Yes	101 (20.2)	39 (7.8)	62 (12.4)	
No	399 (79.8)	313 (62.6)	86 (17.2)	
Breast screening routinely				**<0.001**
Never	449 (89.8)	347 (69.4)	102 (20.4)	
Once a year	37 (7.4)	3 (0.6)	34 (6.8)	
Once every 2 years	8 (1.6)	0 (0.0)	8 (1.6)	
Once every 3 years	4 (0.8)	2 (0.4)	2 (0.4)	
Once every 4 years	2 (0.4)	0 (0.0)	2 (0.4)	
Family history of breast cancer				<**0.001**
Yes	56 (11.2)	31 (6.2)	25 (5.0)	
No	260 (52.0)	166 (33.2)	94 (18.8)	
Don’t know	184 (36.8)	155 (31.0)	29 (5.8)	
Availability of health services for breast screening				**<0.001**
Yes	203 (40.6)	114 (22.8)	89 (17.8)	
No	89 (17.8)	60 (12.0)	29 (5.8)	
Don’t know	208 (41.6)	178 (35.6)	30 (6.0)	
				

*Median (Q1, Q3); Q1 = 1st quartile, Q3 = 3rd quartile, and p-value obtained using Mann-Whitney U test with significance level (α = 0.05). All other values were in frequency and percentages and p-values obtained using a chi-square test with a significance level (α = 0.05).

### 3.3 Description of main TCSB constructs

Table 3 shows the minimum score, maximum score, median, mean, and standard deviation of items associated with the TCSB. To determine these scores, respondents were asked to choose the best level of agreement with each statement from a 7-point Likert scale. For positive statements, strongly agree = 7, agree = 6, slightly agree = 5, neutral = 4 slightly disagree = 3, disagree = 2, and strongly disagree = 7. The reverse score was used for negative statements. For factual statements such as knowledge, a correct response was categorized as low risk, while an incorrect response was categorized as high risk. However, for assessing behaviors such as norms, affect, affirmative responses indicated high norms, affect while those disagreeing with the statements indicated low influences on BCS.

**Table 3:** Description of TCSB constructs.

Variables	Minimum	Maximum	Median	Mean	Std. Dev	Number of Items	Cronbach’s Alpha
Perceived Benefits	2.000	7.000	6.000	5.720	1.093	6	**0.788**
I believe that early screening for breast cancer is necessary for a better disease prognosis	1	7	7	6.390	1.095		
Breast cancer is a very serious health problem that requires regular screening to save lives.	1	7	7	6.300	1.162		
I would rather pray against breast cancer than undergo regular screening	1	7	5	4.740	1.985		
I believe that Allah causes diseases and cures, thus I feel that breast cancer screening is not beneficial.	1	7	6	5.330	1.74		
I believe breast cancer is punishment for disobeying Allah; thus, breast screening is unnecessary	1	7	6	5.900	1.528		
I believe there is anything I can do to prevent breast cancer since Allah is the cause of all diseases.	1	7	6	5.650	1.703		
Knowledge	2.000	7.000	4.375	4.534	0.997	8	**0.694**
I feel I am at a higher risk of getting breast cancer and can access effective treatment	1	7	5	4.42	2.142		
I believe that both prayer to Allah and also medical screening for breast cancer could avert late diagnosis of breast cancer.	1	7	5	4.37	1.984		
I feel I am at a higher risk of getting breast cancer and can readily access screening services	1	7	4	4.6	1.752		
Breast cancer cannot be diagnosed by screening	1	7	5	4.58	1.76		
Breast cancer could be a curse as punishment from Allah as a test of my faith	1	7	4	4.34	1.692		
Offensive blood-stained nipple discharge is a symptom of BC	1	7	4	3.88	1.715		
Breast cancer is hereditary	1	7	5	4.89	1.516		
Itching around the nipples is a symptom of BC	1	7	6	5.2	1.476		
Habits	1.380	7.000	3.938	4.052	1.272	8	**0.813**
Breast screening is necessary even if I have no breast cancer symptoms	1	7	6	5.99	1.443		
I would not undergo screening in the absence of symptoms.	1	7	5.5	4.66	1.965		
I will only get screened when as part of pre-employment medical screening.	1	7	4	4.27	1.954		
I will only undergo breast screening after a health promotion seminar in my community	1	7	4	4.35	1.946		
I will only get screened when I experience offensive nipple discharges.	1	7	4	4.15	2.127		
I will get screened when I experience swelling/thickening of my breasts	1	7	2	3.31	2.13		
Palpating a new lump in the breast or underarm (armpit) will prompt me to screen for BC	1	7	2	2.79	1.888		
Observing flaky skin in the nipple area or the breast would prompt me to screen for BC	1	7	2	2.9	1.927		
Norms	1.000	6.710	4.143	3.951	1.372	7	**0.810**
Male providers at the screening centers violate my religious belief for same-sex healthcare providers	1	7	4	3.71	2.059		
Practices at the screening centers violate my religious values of modesty	1	7	3	3.61	2.07		
Male providers exposing female clients’ bodies at screening centers violate my cultural beliefs.	1	7	5	4.67	2.04		
Exposing my body without the presence of my partner/ husband, brother or an appointed family member violates Islamic religious culture	1	7	5	4.59	2.028		
I need my husband/partner’s approval to undergo breast screening	1	7	4	4.03	2.088		
I will not attend breast screening sessions involving a male provider	1	7	5	4.55	2.061		
I will not attend breast screening sessions involving a female provider	1	7	2	2.5	1.662		
Facilitating Factors	1.600	7.000	5.400	5.212	1.312	5	**0.828**
The proximity of the screening center to my place of residence would facilitate or encourage me to seek BS.	1	7	6	5.63	1.624		
My ability to cover the cost of breast screening would facilitate screening	1	7	6	5.63	1.597		
Permission from my husband/partner would facilitate my BCS	1	7	5	4.49	1.799		
Support from my religious society would facilitate my screening uptake	1	7	6	5.14	1.759		
Support from my community would facilitate my BCS	1	7	6	5.17	1.735		
Affect	1.000	7.000	3.333	3.514	1.687	3	**0.817**
I feel shy undergoing breast cancer screening	1	7	3	3.3	1.967		
I feel embarrassed undergoing breast cancer screening	1	7	3	3.3	1.967		
I am anxious about a possible breast cancer diagnosis	1	7	4	3.94	1.982		
							

The composite median for perceived benefits of screening was 6.0 implying that respondents strongly agreed with statements on benefits. Surprisingly, the median score to the question “I would rather pray against breast cancer than undergo regular screening” was 5 indicating participants slightly agreed with the statement. Moreover, women agreed with the following statements “I believe Allah is the cause of diseases and cure them, thus, breast screening is not beneficial”; “I believe breast cancer is punishment for disobeying Allah; thus, breast cancer screening is unnecessary”; “I believe there is nothing I can do to prevent breast cancer since Allah is the cause of all diseases” indicative of fatalistic tendencies among respondents.

Correctly identifying options about breast cancer knowledge based on the literature was allotted a maximum score of 7. However, the overall median score of 4.375 was observed for the knowledge constructs in Table 3 implying that respondents neither agreed nor disagreed with the knowledge items, and hence were neutral in their responses. Participants scored 4 on the questions “I feel I am at a higher risk of getting breast cancer and can readily access screening services” indicating that participants were neutral. See Table 3 for further details.

### 3.4 Predictors of knowledge of breast cancer

Table 4 is a binary logistic regression model to predict Islamic women’s knowledge of breast cancer. The selection of variables was based on evidence that interaction with HCPs for HIV tests, related problems, or for contraceptive services increased knowledge [47–53]. Moreover, women’s age and parity were excluded from the model as they were strongly correlated with each other indicating the occurrence of multicollinearity. The Nagelkerke R-square obtained was 0.072 which indicates that the variance explained in the dependent variable based on the model is 7.2%.

**Table 4:** Predictors of knowledge breast cancer.

Variables	Unadjusted Odds Ratio (95% CI)	p-value	Adjusted Odds Ratio (95% CI)	p-value
Alcohol consumption				
Never	Reference		Reference	
No, but used to drink	1.09 (0.44-2.74)	0.851	0.86 (0.33-2.24)	0.753
Yes, occasionally at events	0.30 (0.10-0.94)	0.039	0.26 (0.08-0.88)	**0.030**
				
Current use of hormonal contraceptives				
No	Reference		Reference	
Yes	0.85 (0.58-1.24)	0.391	1.17 (0.70-1.96)	0.540
				
Past use of hormonal contraceptives				
No	Reference		Reference	
Yes	0.64 (0.43-0.94)	0.025	0.58 (0.35-0.98)	**0.041**
HIV tests in the past				
Never	Reference		Reference	
Yes, less than 3 months ago	0.45 (0.16-1.27)	0.130	0.40 (0.14-1.15)	0.089
3 months ago,	1.00 (0.26-3.79)	0.998	1.75 (0.40-7.70)	0.462
6 months ago,	1.75 (0.54-5.63)	0.350	1.69 (0.52-5.51)	0.385
Yes, more than 6 months ago	2.50 (1.04-6.02)	0.042	2.30 (0.95-5.57)	0.066
Yes, a year ago	1.80 (0.75-4.35)	0.190	1.75 (0.71-4.32)	0.227
Yes, more than a year ago	1.85 (1.21-2.83)	0.005	1.98 (1.27-3.08)	**0.002**

CI = Confidence Interval

Knowledge of breast cancer was significantly predicted by occasional alcohol consumption, past routine use of contraceptives, and testing for HIV/AIDS. Occasional consumption of alcohol at events was a significantly lower predictor of breast cancer knowledge with 0.26 times lower odds compared to women who had never consumed alcohol (aOR=0.26, 95% CI = 0.08–0.88). A past routine user of contraceptives had 0.58 times lower odds of breast cancer knowledge relative to women who did not use contraceptives in the past (aOR= 0.58, 95% CI = 0.35–0.98). Women who had HIV tests more than a year ago had 1.98 times higher odds of having a higher knowledge of breast cancer compared to women who never had an HIV test done (aOR= 1.98, 95% CI = 1.27–3.08).

### 3.5 Predictors of clinical breast cancer screening

Table 5 presents the predictors of clinical breast cancer screening using binary logistic regression. The binary logistic regression was applied to respondents’ sociodemographic factors, reproductive and lifestyle-related factors as well as the six main constructs of the TCSB. The model shows that of the several factors, seven variables were significantly associated with clinical breast cancer screening. Participant’s level of religiosity had a significant association with breast cancer screening with 1.16 times higher odds of having breast cancer screening as the level of religiosity increased (aOR= 1.16, 95% CI = 1.02–1.32). Additionally, use of contraceptives in the past had 1.93 times higher odds of breast cancer screening compared to women with no history of past contraceptive use (aOR= 1.93, 95% CI = 1.06–3.51). Women who had heard about mammography had 4.43 times the odds of undergoing breast cancer screening compared to those who had not heard of mammography (aOR= 4.43, 95% CI = 2.38–8.25). However, participants who neither knew of their family history of breast cancer (aOR=0.50, 95% CI = 0.28–0.92) nor knew about the availability of health services for breast screening had lower odds of screening (aOR=0.42, 95% CI = 0.20–0.90) compared to women with knowledge of this information. Habits was the only TCSB construct with statistical significance with breast cancer screening (aOR= 1.97, 95% CI = 1.08–3.61; p < 0.028). The Nagelkerke R-square obtained was 0.440 which indicates that the variance explained in the outcome variable based on the model is 44.0%. The details are summarized in Table 5 below.

**Table 5:** Predictors of clinical breast cancer screening.

Variables	Unadjusted Odds Ratio (95% CI)	p-value	Adjusted Odds Ratio (95% CI)	p-value
Age				
18-27	Reference		Reference	
28-39	0.77 (0.48-1.24)	0.281	0.95 (0.40-2.26)	0.907
40-50	0.56 (0.27-1.13)	0.104	0.93 (0.30-2.94)	0.907
51-61	0.85 (0.34-2.13)	0.733	2.75 (0.62-12.19)	0.184
> 61	0.30 (0.04-2.44)	0.258	0.58 (0.03-12.66)	0.730
Income				
less than GH¢366	Reference		Reference	
Between GH¢366–500	0.50 (0.29-0.86)	0.013	0.73 (0.35-1.51)	0.390
Between GH¢ 501–700	0.39 (0.20-0.77)	0.007	0.40 (0.16-1.01)	0.052
Between GH¢701–900	0.63 (0.32-1.23)	0.174	0.39 (0.16-0.94)	**0.036**
GH¢905 and above	0.83 (0.50-1.39)	0.486	0.67 (0.28-1.59)	0.358
Marital Status				
Married	Reference		Reference	
Single	1.73 (1.15-2.60)	0.009	1.20 (0.18-8.14)	0.850
Cohabiting	0.39 (0.05-3.16)	0.374	1.03 (0.06-17.54)	0.984
Widowed	0.49 (0.14-1.72)	0.262	0.58 (0.10-3.25)	0.536
Divorced	0.77 (0.16-3.75)	0.747	1.18 (0.16-8.53)	0.872
Level of Religiosity	1.19 (1.08-1.32)	0.001	1.16 (1.02-1.32)	**0.025**
Level of Education				
No formal education	Reference		Reference	
Primary education	0.30 (0.06-1.53)	0.148	0.49 (0.07-3.32)	0.464
Junior high school/middle school	0.96 (0.27-3.47)	0.95	1.12 (0.23-5.53)	0.893
High school, but did not graduate	0.59 (0.12-3.06)	0.532	0.37 (0.05-2.55)	0.309
High school graduate	1.20 (0.38-3.83)	0.759	0.62 (0.13-2.87)	0.538
HND/Diploma or 3-year degree	2.79 (0.85-9.21)	0.092	1.14 (0.23-5.77)	0.874
4-year college graduate	2.81 (0.87-9.05)	0.083	1.63 (0.34-7.94)	0.545
More than a 4-year college degree	3.17 (0.81-12.42)	0.097	1.42 (0.24-8.38)	0.700
Age of first marriage				
Never been married	Reference		Reference	
< 20 Years Old	0.34 (0.17-0.68)	0.002	0.83 (0.12-5.59)	0.849
≥20 Years Old	0.69 (0.45-1.05)	0.082	1.75 (0.30-10.38)	0.537
Age of menarche	0.97 (0.86-1.09)	0.573	0.88 (0.75-1.03)	0.117
Children				
No	Reference		Reference	
Yes	0.52 (0.35-0.78)	0.001	0.59 (0.23-1.53)	0.277
Past use of hormonal contraceptives				
No	Reference		Reference	
Yes	1.75 (1.16-2.64)	0.008	1.93 (1.06-3.51)	**0.031**
Heard about breast cancer				
No	Reference		Reference	
Yes	2.45 (1.21-4.95)	0.013	2.22 (0.92-5.32)	0.075
Heard about mammography				
No	Reference		Reference	
Yes	5.79 (3.63-9.22)	<0.001	4.43 (2.38-8.25)	**<0.001**
Family history of breast cancer				
No	Reference		Reference	
Yes	1.42 (0.79-2.56)	0.236	1.69 (0.77-3.72)	0.194
Don’t know	0.33 (0.21-0.53)	<0.001	0.50 (0.28-0.92)	**0.025**
Availability of health services for breast screening				
No	Reference		Reference	
Yes	1.62 (0.96-2.73)	0.072	1.28 (0.62-2.63)	0.502
Don’t know	0.35 (0.19-0.63)	<0.001	0.42 (0.20-0.90)	**0.026**
Perceived Benefits				
Low	Reference		Reference	
High	2.05 (1.38-3.04)	<0.001	0.92 (0.50-1.67)	0.776
Knowledge				
Low	Reference		Reference	
High	1.63 (1.10-2.40)	0.014	1.56 (0.91-2.69)	0.107
Habits				
Poor	Reference		Reference	
Good	3.65 (2.41-5.53)	<0.001	1.97 (1.08-3.61)	**0.028**
Norms				
Low	Reference		Reference	
High	0.31(0.21-0.47)	<0.001	0.61(0.34-1.08)	**0.088**
Facilitating Factors				
Poor	Reference		Reference	
Good	0.61 (0.41-0.90)	0.013	0.77 (0.44-1.36)	0.364
Affect				
Low	Reference		Reference	
High	0.73 (0.49-1.07)	0.102	0.84 (0.48-1.47)	0.535
				

CI = Confidence Interval.

## 4 Discussion

Islamic women globally are disproportionately affected by breast cancer [9] and Ghana is no exception[14,36]. This study focused on the factors that influence clinical breast cancer screening among Islamic women in Asokore Mampong Municipality of the Ashanti region of Ghana. Islamic women in the municipality constitute a notable segment of the predominantly Muslim local population[42]. Five hundred participants were selected from the municipality’s high population density, characterized by numerous residents residing in an urban setting, shaped by traditional Islamic values. In this community reproductive health access continues to be shaped by religious and cultural beliefs [54]. The population in the municipality faces socio-economic and healthcare cultural sensitivity challenges that affect women’s access to preventive health screening [54]. The discussion was organized in the order of clinical breast cancer screening participation among participants, followed by the main constructs of the TSCSB such as the social and personal norms, perceived benefits, knowledge, habits, facilitating factors, and affect.

The low CBCS participation (n = 51; 10.2%) obtained in this study is a reflection of the poor state of CBCS in most sub-Saharan African countries where less than a tenth of women undergo CBCS [55]. Authors in Ghana have also reported low screening rates of 10.1% [35], 3.4% [56], 4.5% [6], 9.73% (55), and the highest CBCS prevalence of 18.3% reported among internet users [50]. Results summarized in Table 3 show that participants in this study agreed that personal norms such as “Allah causes diseases and cure, thus breast cancer screening is not beneficial”; “breast cancer is punishment for disobeying Allah; thus, breast cancer screening is unnecessary”; “I do not believe there is anything I can do to prevent breast cancer or cure the disease since Allah is the cause of all diseases and cures them”. Our result is consistent with the work of Shirazi et al [57] in Ghana. Shirazi et al [57] attributed the low breast cancer screening participation among Islamic women to the belief among Islamic women that breast cancer is unavoidable, leading to lower breast screening rates. While Islam emphasizes women’s responsibility to care for their health [58,59], Islamic beliefs might lead to fatalism, limiting women’s influence over breast health [60].

With a median of 9 and 10 for respondents’ level of religiosity and Islamic influence on respondents’ daily decision making respectively indicated that participants in this study were extremely religious population. Religiosity was statistically significant with CBCS as shown in Table 5. The greater a participant’s religiosity the greater the likelihood of undergoing CBCS. Results in Table 3, showed participants indicated that they would refrain from attending CBCS sessions involving male providers. This can be attributed to a male provider exposing female patient bodies in the absence of their partners/husbands, brothers, or appointed family members violated social norms and personal norms. Expectedly, the low CBCS among Islamic women could be attributable to the lack of culturally sensitive screening procedures. This finding is congruent with the results of other studies. Enyan et al [61] contended that the male-provider-dominated healthcare system in Ghana denies Islamic women their right to choose their providers and culturally sensitive care could be reducing Islamic women’s participation in breast cancer screening. Additionally, Ganle [17] and Shirazu et al [57] argued that the religious obligation to maintain bodily sanctity through modest dressing and the avoidance of unlawful bodily exposure or contact, especially with male care providers prevented Islamic women’s participation in CBCS. This outcome is consistent with several studies in Northern Ghana and Nigeria which indicated that women refused healthcare from male nurses due to social norms and traditional prohibitions against male providers exposing female bodies and other sensitive parts [17,62–65].

There was a statistical difference between perceived benefits and breast cancer screening (See Table 1). Even though a significant proportion of women had strong social and personal norms as depicted in the chi-square test of association in Table A1, participants prioritized the benefits of screening over their religious and personal values. This may be due to Islam admonishing its adherents to prioritize health maintenance as Islam ranks health as second to faith [9]. Understandably, the perceived benefit construct had the highest composite median of six relative to the other five TCSB main constructs as shown in Table 3. Despite the high breast cancer awareness and perceived benefits of screening among participants, only one-tenth of the respondents had ever participated in breast cancer screening which is troublesome. From the results in Table 5, the poor CBCS despite women’s high BC awareness and perceived benefits could be due to a lack of cultural competency services during CBCS. In other words, healthcare system-level disregard for women’s personal and social norms during CBCS could have contributed to poor CBCS habits. Thus, women were more likely to defer routine screening only to want to do so symptomatically despite the associated risks linked to such poor habits.

From the chi-square test of association between the main constructs of TCSB and CBCS in Table A1, there was a statistical difference between knowledge about BC and CBCS. This association gives credence to the argument that knowledge about BC is a proxy for CBCS [66]. This is because women with knowledge about BC are more likely to have information about their breast cancer risk and that knowledge might facilitate their active participation in CBCS [67]. Tsegaye et al [68] argued also that women with knowledge about BC were more likely to undertake CBCS because knowledge about BC was more likely to positively impact the acceptability of CBCS programs.

The significantly lower levels of knowledge about BC among respondents in this study are not peculiar to this study but consistent with other studies in Ghana [13,52]. According to Boamah Mensah et al, [69], the low knowledge of BC among rural women was due to the lack of social amenities such as electricity and education about breast cancer. In another study in Ghana, Buunaaim et al. [70] argued that women’s educational attainment and income were critical to participants’ health literacy including knowledge levels about BC. Based on Buunaaim et al.’s [70] argument, the generally low knowledge of participants in this study could be due to women’s low educational attainment. Table 2 shows there was high awareness about breast cancer among the majority of participants. Disappointingly, the high awareness observed did not translate to knowledge about breast cancer among study participants with a composite median of 4 out of 7 (Table 3) as well as a little over half of the participants ranked within the low knowledge level as shown in Appendix A1. Similar studies in Ghana [35,55], India [71], and Tanzania [72] have reported a lack of consistency between high breast cancer awareness and knowledge among women. Women with high knowledge about breast cancer were more likely to undergo screening relative to their counterparts with low knowledge as shown in Table 5. A direct relationship between education and CBCS has been reported by several scholars [69,73–75]. CBCS was statistically significant with educational attainments as shown in Table 1. Evidence has shown that CBCS is a function of women’s employment status and level of education [52,76,77]. A significant proportion of the respondents in this study were unemployed (n = 198; 39.6%) and more than half had only had senior high certificates as their highest educational attainment. Studies have shown that higher CBCS rates correlate with women’s higher educational attainments [76,77]. Educated women were more likely to be aware of the consequences of avoiding preventive screening and could afford the cost of screening relative to women with low educational achievements [76,78].

A majority of women (53%) in this study had an early sexual initiation before the age of 20 years. Authors reported poor socioeconomic conditions such as poverty and low literacy as the causes of women’s early sexual initiation in Ghana [79–82]. The poor screening prevalence coupled with women’s decreased likelihood to afford CBCS services in a population with a genetic predisposition to estrogen receptor (HER2) negative cancers is concerning [1,83]. Participants’ monthly income was statistically significant with CBCS as shown in Table 1. Earning Gh¢701–900 was statistically significant with CBCS (Table 5). Participants with Gh ¢701–900 as monthly income had a lower likelihood of screening than their counterparts who earned less. In other words, the greater the monthly earnings of respondents, the higher the likelihood of screening. It appears that participants’ monthly income provided increased financial access to CBCS and thus was a critical determinant of BCS. This outcome is consistent with earlier reports from scholars in Ghana [6,52,55,56,74,84].

Knowledge was a statistically significant predictor of breast cancer screening. Knowledge about breast cancer is transmitted to women through interactions with HCPs. Frequent healthcare encounters between patients and HCPs have been associated with increased knowledge in several studies [47–53]. Table 4 shows interactions between patients and HCPs were assessed for their impact on women’s breast cancer knowledge. Of the several factors, alcohol consumption, history of past contraceptive use, and HIV screening significantly impacted women’s knowledge of breast cancer. Due to the prohibition of alcohol consumption by Islam, overall, less than two-fifths of participants either consumed alcohol or were past consumers of alcohol (Table 2).

History of past hormonal contraceptive use was a significant predictor of knowledge of breast cancer. There was a significant statistical association between past users of contraceptives and women who had never used contraceptives. Past users were less likely to be knowledgeable about breast cancer relative to women with no history of use. This gives cause for concern given the evidence of a 20–30% higher risk of breast cancer for a 5-year use of hormonal contraceptives [85]. A study in Ghana attributed the low breast cancer knowledge to the lack of education by clinicians who cited heavy workloads as the main barrier to their educational role [13]. History of past hormonal contraceptive use was significantly associated with CBCS. Results in Table 5 demonstrated women with a history of past hormonal contraceptive use had a greater likelihood of screening relative to their counterparts without a history of use. Due to the lack of nationwide implementation of breast cancer screening programs in many African countries including Ghana, breast cancer screening could be largely opportunistic [13,86]. Table 5 presents the impact of the opportunistic interactions between respondents and their HCPs. The greater likelihood of screening among past hormonal contraceptive users could be due to two reasons. First, although routine CBCS may not be universally mandated before hormonal contraceptives [87], but owing to varying hospital practices and opportunistic screening due to the lack of a comprehensive nationwide breast cancer screening policy in Ghana, it is possible that clinicians may be prescribing CBCS during contraceptive service provision. Secondly, it is plausible that participants who were past contraceptive users were exposed to education and counseling on CBCS during contact with the HCP during contraceptive service provision.

The low mammography awareness among participants (89.8%) in this study is problematic as poor mammography awareness has implications for the early detection and treatment efforts of breast cancer in Ghana. In line with Ghana’s annual screening campaigns every October, it is reported that the Ghana Health Service—the government body in charge of delivering comprehensive health services—nongovernmental organizations (NGOs), as well as health professional groups and associations, recommend mammography at the age of 40 years [12]. However, there is a paucity of robust or accessible proactive screening programs targeting the general population [55]. A lack of comprehensive breast cancer surveillance and awareness systems for women at age 40 in Ghana, coupled with the fact that the majority of the women in this study were less than the eligible age for mammography examination could have limited women’s awareness. Sekar et al [71], argued that the lack of congruence between increased breast cancer awareness and screening behavior indicated that other barriers impede women from getting proper screening including economic resources, such as education and health literacy.

Evidence shows a direct link between women’s educational attainments and mammography awareness [13,55,88,89]. Women with higher educational attainment had higher mammography awareness than those with lower educational attainment [88,89]. For instance, according to a study carried out in Ethiopia, about 83% of breast cancer patients with no education had no awareness of mammography [88]. Comparatively, awareness of 99% was reported in an Indian study involving women with tertiary-level education [89]. Duguma et al.[88] argued that due to the positive correlation between educational attainments and mammography awareness women who had completed secondary education were 4.5 times more likely to be aware of mammography compared to women with no formal education. However, a 5.7 times greater likelihood of mammography awareness was reported for women with tertiary-level education relative to those with no formal education [88]. The lower than tertiary-level education among most respondents in this study could have lowered participants’ health literacy, which is associated with more proactive health behaviors, including mammography awareness [90]. Studies in Ghana have reported similarly low mammography awareness among women[14,55,91,92]. Unsurprisingly, women with awareness of mammography in this study were more than four times more likely to undergo breast cancer screening relative to their counterparts who had not heard about mammography as shown in Table 5. This could be due to several reasons. First, notwithstanding the high perceived benefits of screening observed in this study in addition to the direct association between higher educational levels and mammography awareness [88]. Given the limited locations of mammography centers in Ghana [14], the age of 40 years as the age of eligibility for mammography screening coupled with the poor mammography services utilization in Ghana [15], it is possible that participants with higher mammography awareness could be older women with higher education levels and access to health information. Older age, higher education, and health literacy have been linked to more proactive health behaviors such as mammography awareness and mammography screening participation [90,93]. Secondly, given the scarcity of mammography services and the age of 40 as the eligibility age for screening in Ghana, some researchers have argued that women who had heard about mammography might have heard about the examination from interactions with their providers [14,91]. Provider recommendations can significantly lower women’s psychological barriers to using mammography, and shape attitudes and social norms that influence the decision to undergo breast cancer screening [91].

The habit construct was statistically significant with CBCS from the chi-square test of association (S1 Appendix). There was a split between women with good habits and those with poor CBCS habits. Based on the composite score of 3.9 (Table 3), the majority of responses for habits in this study indicated that participants would undergo CBCS only symptomatically. This outcome is a confirmation of evidence that Ghanaian women generally lacked the attitude of habitually seeking CBCS in the absence of breast symptoms [13,52]. Screening symptomatically is an issue given Ghana’s limited human and material resources for the care of breast cancer patients [13,14,36]. Deferring screening until symptom presentation is a poor health habit with a tendency for advanced breast cancer diagnosis and a poor survival rate for women [14]. Boamah Mensah et al.[69] attributed the symptomatic screening among women in Ghana to the shortage of public walk-in breast screening centers for the majority of women in Ghana. Moreover, the limited breast cancer screening clinics in Ghana were described as breast pathology clinics, not walk-in breast screening clinics. Thus, the limited screening facilities delayed women’s screening [13]. Women with the belief that asymptomatic screening was necessary were more likely to seek screening relative to their counterparts who would only screen upon the presentation of breast cancer symptoms.

Facilitating factors were not statistically significant with breast cancer screening (Table 5). Generally, respondents agreed that the proximity of screening centers to their places of residence, their ability to cover the cost of screening, their partner/husband’s support for screening, and support from community and religious groups would facilitate their screening (Table 3). The majority of women in this study had good facilitating factors (Table A1) but were less likely to screen (Table 5). Regardless of whether facilitating factors were good or poor, almost the same number of women with neither good nor poor facilitating factors had not screened. Surprisingly, a greater number of participants with poor facilitating factors had screened compared to women with good facilitating factors (Table A1). The strong inclination to screen despite poor facilitating factors could likely be due to the high perceived benefits of CBCS as shown in Table 3. Results showed that women had a strong agreement with the perceived benefits construct (composite median of 6; Table 3), but Ghana’s lack of a nationwide screening program that is uniquely designed for Islamic women may have restricted participation. Moreover, the poor coordination of community-level awareness and activation of facilitating factors for sustained education on BC [13] may have resulted in the poor CBCS observed in this study. Ghana lacks screening facilities and systematic nationwide screening programs that include CBCS and mammography for women who are willing to screen [10,13,94]. Despite evidence that screening facilities are situated in urban areas of Ghana [13], a significant majority of women in this study lacked knowledge of the availability of screening facilities (Table 2). The lack of knowledge about the availability of screening facilities could have implications for early BC detection in Ghana [13]. It is plausible that patients would experience poorer outcomes if the disease was detected at a later stage. Women with no knowledge of the availability of health services for breast screening had over 40% less likelihood of screening relative to participants with no availability of health services for CBCS. Meanwhile, women with knowledge of available healthcare services for CBCS were more likely to screen compared to their counterparts with no knowledge. These differences in the degree of screening possibilities could be the prioritization of CBCS. Despite the lack of statistical difference between women with no knowledge about available resources and those with knowledge of available resources, women with knowledge of available resources for screening had greater odds of screening than their counterparts who did not know about available community resources for screening.

### 4.1 Strengths and limitations

This study’s strength is found in its diverse respondent sample. Also, to the extent of our knowledge, this is the first study to assess the factors that influence CBCS among Islamic women in Ghana to inform healthcare decision-making in this demographic and serve as the basis for future studies.

One of the study’s limitations is that it was carried out in only one of the many Islam-populated areas in Kumasi, Ghana thus the findings are not nationally applicable. Furthermore, the cross-sectional systematic design deployed in the study does not establish causal links.

This study did not compare the factors influencing CBCS across the two communities from which the study was drawn. Although the differences in ethnic composition between the two communities may have yielded additional or different results, no comparison of the factors influencing Islamic women’s different breast cancer screening behavior was conducted. This is because the study focused on measuring the factors that influence breast cancer screening broadly in the two ethnically diverse communities in the Asokore Mampong Municipality.

The addition of women of other faiths may have provided additional themes. The exclusive of populations of other faiths limits the generalizability of the results to the entire population of women in the municipality.

## 5 Conclusion

The study concludes that despite the perceived high benefits of clinical breast cancer screening, actual clinical breast cancer screening practices were poor due to fear of unmet cultural preferences at screening centers and poor knowledge about breast cancer. This implies that this population subtype could present late to screening centers with breast cancer disease in advanced stages associated with increased mortality. Thus, implementing national breast cancer screening campaigns that emphasize the need for asymptomatic (routine) clinical breast cancer screening and providing culturally sensitive screening practices cannot be overemphasized.

## Supporting information

S1 Data Questionnaire. Contains question items that were used for the study. (DOCX)

S1 Appendix Table A1: Association Between Breast Cancer Screening and the Constructs of TCSB. (DOCX)
