# Supplementary material for: Factors influencing clinical breast cancer screening: A cross-sectional study among Islamic women in Kumasi Metropolis of Ghana
Source: PLoS One. 2025 May 23;20(5):e0320726. doi: 10.1371/journal.pone.0320726 (PMC12101858; doi:10.1371/journal.pone.0320726)
Supplement: S1 Data — (DOCX) [file pone.0320726.s001.docx]

**FACTORS INFLUENCING CLINICAL BREAST CANCER SCREENING AMONG ISLAMIC WOMEN IN THE KUMASI METROPOLIS OF GHANA.**

**SECTION A: SOCIO-DEMOGRAPHIC INFORMATION (About You)**

***Mark only one oval***

1. What is your current age (18+)_____________________________________
2. What is your level of income?

**Ο** less than GH¢366 **Ο** Between GHC 366-500 **Ο** Between GH¢ 501-700

**Ο** Between GH¢701-900 **Ο** Between GHC905 and above

1. Marital Status

**Ο** Married (Answer Q.4) **Ο** Single (Skip Q. 4) **Ο** Cohabiting (Skip Q.4)

**Ο** Widowed (Answer Q.4) **Ο** Divorced (Answer Q.4)

1. Would you describe your present or past marriage as polygamous?

**Ο** Yes **Ο** No

1. What sect of Islam are you affiliated to?

**Ο** Orthodox **Ο** Ahlus Sunna Wal-Jama'a **Ο** Tijaniyya **Ο** Option 4

**Ο** Ahmadiyya **Ο** Shia **Ο** Other: ____________________________________

1. Using any number from 0 to 10, where 0 means that you are not religious at all and 10 is the maximum level of religiosity, what number would you use to rate how much of a religious person you are?

| Not a religious person | **0** | **1** | **2** | **3** | **4** | **5** | **6** | **7** | **8** | **9** | **10** | Completely a religious person |
| --- | --- | --- | --- | --- | --- | --- | --- | --- | --- | --- | --- | --- |
|  | **Ο** | **Ο** | **Ο** | **Ο** | **Ο** | **Ο** | **Ο** | **Ο** | **Ο** | **Ο** | **Ο** |  |

1. Using any number from 0 to 10, where 0 means that religion does not play a role in your life at all and 10 means that religion plays a major role in your life, what number would you use to rate how much religion plays a role in your life?

| Religion does not play a role in your life at a | **0** | **1** | **2** | **3** | **4** | **5** | **6** | **7** | **8** | **9** | **10** | Religion plays a major role in your life |
| --- | --- | --- | --- | --- | --- | --- | --- | --- | --- | --- | --- | --- |
|  | **Ο** | **Ο** | **Ο** | **Ο** | **Ο** | **Ο** | **Ο** | **Ο** | **Ο** | **Ο** | **Ο** |  |

1. Which among the following represents your Social Class?

**Ο** Upper Middle-Class **Ο** Middle-Class **Ο** Lower Middle-Class **Ο** Skilled Working Class

**Ο** Working Class **Ο** Non-working Class

1. Employment Status

**Ο** Self-employed **Ο** Unemployed **Ο** Government employee **Ο** Student **Ο** Housewife **Ο** Retired

**Ο** Private sector employee

1. What is your occupation?

**Ο** Petty trading **Ο** Fishmonger **Ο** Dressmaking **Ο** Farmer **Ο** Artisan **Ο** Professional

**Ο** Hairdressing **Ο** Student without an occupation

1. What is the highest grade or level of education you received?

**Ο** No formal education **Ο** Primary education **Ο** Junior high school/middle school

**Ο** High school, but did not graduate **Ο** High school graduate **Ο** HND/Diploma or 3-year degree

**Ο** 4-year college graduate **Ο** More than 4-year college degree

1. What is your ethnic affiliation? Please mark one or more.

**Ο** Northern descent **Ο** Asante **Ο** Akan tribe descent other than Asante **Ο** Ga **Ο** Ewe

**Ο** non-Ghanaian **Ο** Other: _________________________________________________________________

1. Are you a migrant from outside of Ghana or within Ghana?

**Ο** Yes, a migrant from within Ghana **Ο** No, a migrant from outside Ghana **Ο** A native of Asante region **Ο** Other: _________________________________________

1. Did someone help you complete this survey?

**Ο** Yes **Ο** No

1. How did that person help you? Mark all that apply

*Mark all that apply * Check all that apply.*

**Ο** Read the questions to me **Ο** Wrote down the answers I gave **Ο** Answered the questions for me

**Ο** Translated the questions into my language **Ο** Helped in some other way (Please print)

**SECTION B: LIFESTYLE**

1. Smoking

**Ο** Never **Ο** No, but a former smoker **Ο** Yes, occasionally at events **Ο** Yes, daily **Ο** Yes, once a week

1. Types of foods consumed? (**Select all that apply**)

*Check all that apply.*

**Ο** Fast foods **Ο** Fruits and vegetables **Ο** Local dishes **Ο** Mainly fast foods and canned foods

1. How often do you eat fruits and vegetables?

**Ο** Never **Ο** 1-2 times per week **Ο** 3-4 times per week **Ο** 5-6 times per week **Ο** 7 or more times per week **Ο** I do not know

1. Alcohol consumption?

**Ο** Never Ο No, but I used to drink **Ο** Yes, occasionally at events **Ο** Yes, daily **Ο** Yes, once a week

1. Exercise regularly

**Ο** Yes, I do at least 30 minutes of moderate-intensity aerobic activity 5 days a week.

**Ο** Yes, I do at least 30 minutes of moderate-intensity aerobic activity less than 5 days a week

**Ο** Yes, I do brisk walking exercise occasionally **Ο** No, not at all

**SECTION C: HYGIENE BEHAVIOR**

1. Bathing Daily

**Ο** Yes, once a day **Ο** Yes, twice a day **Ο** Yes, more than twice a day **Ο** No

1. Water Source for Washing Clothes

**Ο** Driven or drilled well **Ο** Tap water Ο River **Ο** Dug Well

**SECTION D: REPRODUCTIVE HISTORY**

1. Age of First Marriage

**Ο** < 20 Years Old **Ο** ≥20 Years Old **Ο** Never been married

1. Age of first menstruation (Menarche)____________________________________
2. Do you have a child or children?

**Ο** Yes (If yes go to Questions 27 & 28) **Ο** No (If no skip to Question 28)

1. What age did you deliver your first child? ____________________________________________
2. How many children do you have?

**Ο** None **Ο** 1 **Ο** 2 **Ο** 3 **Ο** 4 or above

1. Which age was your first sexual debut?

**Ο** Never been sexually exposed **Ο** Below 15 **Ο** 15-20 **Ο** 21-25 **Ο** 26-30 **Ο** 31-40 **Ο** Above 40

1. Have you had an HIV test in the past?

**Ο** Never **Ο** Yes, less than 3 months ago Ο 3 months ago Ο 6 months ago Ο Yes, more than 6 months ago, Ο Yes, a year ago **Ο** Yes, more than a year ago

|  | Yes | No |
| --- | --- | --- |
| 1. Routine use of contraceptives including emergency contraceptives such as Depot, Combined oral contraceptives, injectables, postinor 2, Lydia, etc. presently?) | **Ο** | **Ο** |
| 1. Routine use of contraceptives including emergency contraceptives such as Depot, Combined oral contraceptives, injectables, postinor 2, Lydia, etc. in the past? | **Ο** | **Ο** |
| 1. Lump in your breast? | **Ο** | **Ο** |

**SECTION E: HEALTH SERVICES AND CANCER PREVENTION BEHAVIOR**

|  | Yes | No |
| --- | --- | --- |
| 1. Have you heard of breast cancer disease? | **Ο** | **Ο** |
| 1. Have you had breast cancer screening? | **Ο** | **Ο** |
| 1. Heard of mammography Tests? | **Ο** | **Ο** |

1. Have you undergone breast screening routinely?

**Ο** Once a year **Ο** Once every 2 years **Ο** Once every 3 years **Ο** Once every 4 years

**Ο** I screen at will **Ο** Never

|  | Yes | No | I don't know |
| --- | --- | --- | --- |
| 1. Family History of breast cancer (BC)? | **Ο** | **Ο** | **Ο** |
| 1. Health facilities within 5 km of the residence? | **Ο** | **Ο** | **Ο** |
| 1. Are Health services available for breast screening? | **Ο** | **Ο** | **Ο** |

**SECTION F: FATALISM/BENEFITS (To what extent do you agree/disagree with the following statements concerning breast screening?)**

|  | Strongly Agree | Agree | Slightly Agree | Neutral | Slightly Disagree | Disagree | Strongly Disagree |
| --- | --- | --- | --- | --- | --- | --- | --- |
| 1. I believe that early screening for breast cancer is necessary for a better disease prognosis | **Ο** | **Ο** | **Ο** | **Ο** | **Ο** | **Ο** | **Ο** |
| 1. Breast cancer is a very serious health problem that requires regular screening to save lives. | **Ο** | **Ο** | **Ο** | **Ο** | **Ο** | **Ο** | **Ο** |
| 1. I would rather pray against breast cancer than undergo regular screening | **Ο** | **Ο** | **Ο** | **Ο** | **Ο** | **Ο** | **Ο** |
| 1. I believe that Allah causes diseases and cures, thus I feel that breast screening is not beneficial. | **Ο** | **Ο** | **Ο** | **Ο** | **Ο** | **Ο** | **Ο** |
| 1. I believe breast cancer is punishment for disobeying Allah, thus breast screening is unnecessary | **Ο** | **Ο** | **Ο** | **Ο** | **Ο** | **Ο** | **Ο** |
| 1. I do not believe there is anything I can do to prevent breast cancer or cure the disease since Allah is the cause of all diseases and cures them. | **Ο** | **Ο** | **Ο** | **Ο** | **Ο** | **Ο** | **Ο** |

**SECTION G: KNOWLEDGE (This section assesses your knowledge about breast cancer)**

*(Strongly Agree, Agree, Slightly Agree, Neutral, Slightly Disagree, Disagree, Strongly Disagree)*

|  | Strongly Agree | Agree | Slightly Agree | Neutral | Slightly Disagree | Disagree | Strongly Disagree |
| --- | --- | --- | --- | --- | --- | --- | --- |
| 1. I feel I am at a higher risk of getting breast cancer and can access effective treatment | **Ο** | **Ο** | **Ο** | **Ο** | **Ο** | **Ο** | **Ο** |
|  |  |  |  |  |  |  |  |
| 1. I believe that both prayer to Allah and also medical screening for breast cancer could avert late diagnosis of breast cancer. | **Ο** | **Ο** | **Ο** | **Ο** | **Ο** | **Ο** | **Ο** |
| 1. I feel I am at a higher risk of getting breast cancer and can readily access screening services | **Ο** | **Ο** | **Ο** | **Ο** | **Ο** | **Ο** | **Ο** |
| 1. Breast cancer cannot be diagnosed by screening? | **Ο** | **Ο** | **Ο** | **Ο** | **Ο** | **Ο** | **Ο** |
| 1. Breast cancer could be a curse as punishment from Allah as a test of Breast cancer could be a curse as punishment from Allah as a test of | **Ο** | **Ο** | **Ο** | **Ο** | **Ο** | **Ο** | **Ο** |
| 1. Offensive blood-stained nipple discharge is a symptom of BC | **Ο** | **Ο** | **Ο** | **Ο** | **Ο** | **Ο** | **Ο** |
| 1. Breast cancer is hereditary | **Ο** | **Ο** | **Ο** | **Ο** | **Ο** | **Ο** | **Ο** |
| 1. Itching around the nipples is a symptom of BC | **Ο** | **Ο** | **Ο** | **Ο** | **Ο** | **Ο** | **Ο** |
|  | **Ο** | **Ο** | **Ο** | **Ο** | **Ο** | **Ο** | **Ο** |
|  | **Ο** | **Ο** | **Ο** | **Ο** | **Ο** | **Ο** | **Ο** |
|  | **Ο** | **Ο** | **Ο** | **Ο** | **Ο** | **Ο** | **Ο** |
|  | **Ο** | **Ο** | **Ο** | **Ο** | **Ο** | **Ο** | **Ο** |

*(Very Poor, Poor, Fair, Good, Very good, Excellent)*

|  | Very Poor | Poor | Fair | Good | Very good | Excellent |
| --- | --- | --- | --- | --- | --- | --- |
| 1. How would you rate your knowledge of breast cancer? | **Ο** | **Ο** | **Ο** | **Ο** | **Ο** | **Ο** |
| 1. How would you rate your breast screening behavior? | **Ο** | **Ο** | **Ο** | **Ο** | **Ο** | **Ο** |
| 1. In general, how would you rate your overall health? | **Ο** | **Ο** | **Ο** | **Ο** | **Ο** | **Ο** |

**SECTION H: HABITS (Tell us the extent to which you are likely to screen with or without symptoms)**

*(Strongly Agree, Agree, Slightly Agree, Neutral, Slightly Disagree, Disagree, Strongly Disagree)*

|  | Strongly Agree | Agree | Slightly Agree | Neutral | Slightly Disagree | Disagree | Strongly Disagree |
| --- | --- | --- | --- | --- | --- | --- | --- |
| 1. Breast screening is necessary even if I have no breast cancer symptoms | **Ο** | **Ο** | **Ο** | **Ο** | **Ο** | **Ο** | **Ο** |
| 1. I would not undergo screening in the absence of symptoms. | **Ο** | **Ο** | **Ο** | **Ο** | **Ο** | **Ο** | **Ο** |
| 1. I will only get screened when as part of pre-employment medical screening. | **Ο** | **Ο** | **Ο** | **Ο** | **Ο** | **Ο** | **Ο** |
| 1. I will only undergo breast screening after a health promotion seminar in my community | **Ο** | **Ο** | **Ο** | **Ο** | **Ο** | **Ο** | **Ο** |
| 1. I will only get screened when I experience offensive nipple discharges. | **Ο** | **Ο** | **Ο** | **Ο** | **Ο** | **Ο** | **Ο** |
| 1. I will get screened when I experience swelling/thickening of my breasts | **Ο** | **Ο** | **Ο** | **Ο** | **Ο** | **Ο** | **Ο** |
| 1. Palpating a new lump in the breast or underarm (armpit) will prompt me to screen for BC | **Ο** | **Ο** | **Ο** | **Ο** | **Ο** | **Ο** | **Ο** |
| 1. Observing flaky skin in the nipple area or the breast would prompt me to screen for BC | **Ο** | **Ο** | **Ο** | **Ο** | **Ο** | **Ο** | **Ο** |

**SECTION I: NORMS (To what extent do you agree or disagree with the following statements)**

*(Strongly Agree, Agree, Slightly Agree, Neutral, Slightly Disagree, Disagree, Strongly Disagree)*

|  | Strongly Agree | Agree | Slightly Agree | Neutral | Slightly Disagree | Disagree | Strongly Disagree |
| --- | --- | --- | --- | --- | --- | --- | --- |
| 1. Male providers at the screening centers violate my religious belief for same-sex healthcare providers | **Ο** | **Ο** | **Ο** | **Ο** | **Ο** | **Ο** | **Ο** |
|  | | | | | | | |
| 1. Practices at the screening centers violate my religious values of modesty | **Ο** | **Ο** | **Ο** | **Ο** | **Ο** | **Ο** | **Ο** |
| 1. Male providers exposing female clients' bodies at screening centers violate my cultural beliefs. | **Ο** | **Ο** | **Ο** | **Ο** | **Ο** | **Ο** | **Ο** |
| 1. Exposing my body without the presence of my partner/ husband, brother or an appointed family member violates Islamic religious culture | **Ο** | **Ο** | **Ο** | **Ο** | **Ο** | **Ο** | **Ο** |
| 1. I need my husband/partner’s approval to undergo breast screening | **Ο** | **Ο** | **Ο** | **Ο** | **Ο** | **Ο** | **Ο** |

**SECTION J: FACILITATING FACTORS (Factors that you can think would enhance your uptake of breast screening)**

*(Strongly Agree, Agree, Slightly Agree, Neutral, Slightly Disagree, Disagree, Strongly Disagree)*

|  | Strongly Agree | Agree | Slightly Agree | Neutral | Slightly Disagree | Disagree | Strongly Disagree |
| --- | --- | --- | --- | --- | --- | --- | --- |
| 1. The proximity of the screening center to my place of residence would facilitate or encourage me to seek BS. | **Ο** | **Ο** | **Ο** | **Ο** | **Ο** | **Ο** | **Ο** |
| 1. My ability to cover the cost of breast screening would facilitate screening | **Ο** | **Ο** | **Ο** | **Ο** | **Ο** | **Ο** | **Ο** |
| 1. Requires my husband/partner's permission to undertake screening would facilitate my screening | **Ο** | **Ο** | **Ο** | **Ο** | **Ο** | **Ο** | **Ο** |
| 1. Support from my religious society would facilitate my screening uptake | **Ο** | **Ο** | **Ο** | **Ο** | **Ο** | **Ο** | **Ο** |
| 1. Support from my community would facilitate my BS uptake | **Ο** | **Ο** | **Ο** | **Ο** | **Ο** | **Ο** | **Ο** |

**SECTION K: AFFECT (To what extent would you be encouraged or discouraged to get screened by the following circumstances), (Which of the following are likely to hinder you from seeking clinical breast screening)**

*(Strongly Agree, Agree, Slightly Agree, Neutral, Slightly Disagree, Disagree, Strongly Disagree)*

|  | Strongly Agree | Agree | Slightly Agree | Neutral | Slightly Disagree | Disagree | Strongly Disagree |
| --- | --- | --- | --- | --- | --- | --- | --- |
|  |  |  |  |  |  |  |  |
|  |  |  |  |  |  |  |  |
| 1. Feel shy or embarrassed undergoing Breast Screening | **Ο** | **Ο** | **Ο** | **Ο** | **Ο** | **Ο** | **Ο** |
| 1. I fear of losing my virginity when I undergo breast screening | **Ο** | **Ο** | **Ο** | **Ο** | **Ο** | **Ο** | **Ο** |
| 1. I am anxious about a possible breast cancer diagnosis | **Ο** | **Ο** | **Ο** | **Ο** | **Ο** | **Ο** | **Ο** |

1. How often have you sought clinical breast screening for yourself in the last 12 months?

**Ο** Never (End of survey, Thank you for your time)

**Ο** Rarely (Please answer the Questions that follow)

**Ο** Occasionally (Please answer the Questions that follow)

**Ο** Sometimes (Please answer the Questions that follow)

**Ο** Regularly (Please answer Questions that follow)

1. What types of breast screening have you undergone in the past? (Please select as many as apply)

**Ο** Clinical breast exam **Ο** Digital Breast Tomosynthesis (3-D Mammography)

**Ο** Digital Mammography Ο Breast Ultrasound **Ο** Breast MRI Ο Breast Needle Biopsy

**Ο** Cyst or Fine Needle Aspiration **Ο** Contrast-enhanced digital Mammography (CEDM)

|  | Yes | No |
| --- | --- | --- |
| 1. A personal healthcare provider/doctor is the one you would see if you need a screening, want advice about a health problem, or get sick or hurt. Do you have a specific provider for breast cancer screening (BCS)? | **Ο** | **Ο** |
| 1. Please think of the last provider as you answer the following questions. Is this the provider you usually see if you need breast screening (BS), and want education and treatment options for breast cancer (BC)? | **Ο** | **Ο** |

1. How long have you been going to this provider?

**Ο** Less than 6 months **Ο** At least 6 months but less than 1 year **Ο** At least 1 year but less than 3 years

**Ο** At least 3 years but less than 5 years **Ο** 5 years or more

**L. HEALTH CARE PROVIDER (HCP)**

**PROVIDER CULTURAL COMPETENCY FROM THE PATIENT'S PERSPECTIVE.**

Please indicate how often each statement applies to your situation by selecting one option.

Your care during visits to your Provider. These questions ask about your health care. Do not include the care you got when you stayed overnight in a hospital. Do not include the times you went for dental care visits.

**SECTION K: PROVIDER COMMUNICATION**

*(Never, Rarely, Sometimes, Usually, Occasionally, Always)*

|  | Never | Rarely | Sometimes | Usually | Occasionally | Always |
| --- | --- | --- | --- | --- | --- | --- |
| 1. My HCP explains things in a way that is easy to understand. | **Ο** | **Ο** | **Ο** | **Ο** | **Ο** | **Ο** |
| 1. My HCP listens carefully to me. | **Ο** | **Ο** | **Ο** | **Ο** | **Ο** | **Ο** |
| 1. My HCP spends enough time with me. | **Ο** | **Ο** | **Ο** | **Ο** | **Ο** | **Ο** |
| 1. My HCP interrupts me when I am talking. | **Ο** | **Ο** | **Ο** | **Ο** | **Ο** | **Ο** |
| 1. My HCP talks too fast when talking with me. | **Ο** | **Ο** | **Ο** | **Ο** | **Ο** | **Ο** |
| 1. My HCP uses medical words I do not understand. | **Ο** | **Ο** | **Ο** | **Ο** | **Ο** | **Ο** |
| 1. The HCP gives explanations that are too hard to understand because of an accent or the way they spoke English. | **Ο** | **Ο** | **Ο** | **Ο** | **Ο** | **Ο** |
| 1. My HCP ignores what I told him/her. | **Ο** | **Ο** | **Ο** | **Ο** | **Ο** | **Ο** |
| 1. My HCP uses a condescending, sarcastic, or rude tone or manner with me. | **Ο** | **Ο** | **Ο** | **Ο** | **Ο** | **Ο** |
| 1. My HCP shows interest in my questions and concerns. | **Ο** | **Ο** | **Ο** | **Ο** | **Ο** | **Ο** |
| 1. My provider answers all my questions to my satisfaction. | **Ο** | **Ο** | **Ο** | **Ο** | **Ο** | **Ο** |
| 1. I have had talks with my provider on minimizing my risks, treatment options, breast cancer, and screening for breast cancer. | **Ο** | **Ο** | **Ο** | **Ο** | **Ο** | **Ο** |
| 1. My HCP gives me easy-to-understand instructions about breast cancer care. | **Ο** | **Ο** | **Ο** | **Ο** | **Ο** | **Ο** |
| 1. My HCP talks about breast self-examination 3 to 5 days after my monthly period starts. | **Ο** | **Ο** | **Ο** | **Ο** | **Ο** | **Ο** |
| 1. People sometimes see someone else besides their providers or specialists to help with an illness or to stay healthy. In the last 12 months, I have used the services of a herbalist or a traditional priest. | **Ο** | **Ο** | **Ο** | **Ο** | **Ο** | **Ο** |
| 1. Has your HCP ever asked you if you have used the services of alternative health practitioners to help with an illness or to stay healthy (for example, a Fetish priest, herbalist, pastor, or Islamic cleric)? | **Ο** | **Ο** | **Ο** | **Ο** | **Ο** | **Ο** |

1. Using any number from 0 to 10, where 0 is the worst doctor possible and 10 is the best doctor possible, what number would you use to rate this doctor?

| Worst Doctor Possible | **0** | **1** | **2** | **3** | **4** | **5** | **6** | **7** | **8** | **9** | **10** | Best Doctor possible |
| --- | --- | --- | --- | --- | --- | --- | --- | --- | --- | --- | --- | --- |
|  | **Ο** | **Ο** | **Ο** | **Ο** | **Ο** | **Ο** | **Ο** | **Ο** | **Ο** | **Ο** | **Ο** |  |

**II. PROVIDER SHARED DECISION MAKING (SDM)**

**Patient's perspective of provider-shared decision-making**

*(Never, Rarely, Sometimes, Usually, Occasionally, Always)*

|  | Never | Rarely | Sometimes | Usually | Occasionally | Always |
| --- | --- | --- | --- | --- | --- | --- |
| 1. Choices for your treatment or health care can include choices about chemotherapy, surgery, or radiotherapy treatment. In the last year, did your provider tell you there was more than one choice for breast cancer care? | **Ο** | **Ο** | **Ο** | **Ο** | **Ο** | **Ο** |
| 1. Have you had conversations with your provider concerning the pros and cons of each choice of treatment options for breast cancer in case you are diagnosed? | **Ο** | **Ο** | **Ο** | **Ο** | **Ο** | **Ο** |
| 1. When there was more than one choice for your treatment or health care, did your provider ask which choice you thought was best for you? | **Ο** | **Ο** | **Ο** | **Ο** | **Ο** | **Ο** |
| 1. I prefer that my provider asks my opinion about the choices that I believe are best for me. | **Ο** | **Ο** | **Ο** | **Ο** | **Ο** | **Ο** |
| 1. I prefer to leave decisions about my treatment or breast cancer check up to my HCP. | **Ο** | **Ο** | **Ο** | **Ο** | **Ο** | **Ο** |

**III. EXPERIENCE AT THE PROVIDERS OFFICE**

**Patients’ experiences at the providers’ office**

*(Never, Rarely, Sometimes, Usually, Occasionally, Always)*

|  | Never | Rarely | Sometimes | Usually | Occasionally | Always |
| --- | --- | --- | --- | --- | --- | --- |
| 1. Do you ever feel that you have been treated unfairly at your HCP's office because of your religion (Islam)? | **Ο** | **Ο** | **Ο** | **Ο** | **Ο** | **Ο** |
| 1. 114. Do you ever feel that you have been treated unfairly at your HCP's office because of your ethnicity/tribe? | **Ο** | **Ο** | **Ο** | **Ο** | **Ο** | **Ο** |

**IV. TRUST**

**Patients Level of trust for the provider**

*(Never, Rarely, Sometimes, Usually, Occasionally, Always)*

|  | Never | Rarely | Sometimes | Usually | Occasionally | Always |
| --- | --- | --- | --- | --- | --- | --- |
| 1. Do you feel you can tell your HCP anything, even things that you might not tell anyone else? | **Ο** | **Ο** | **Ο** | **Ο** | **Ο** | **Ο** |
| 1. Do you trust your HCP with your breast cancer screening | **Ο** | **Ο** | **Ο** | **Ο** | **Ο** | **Ο** |
| 1. Do you feel your HCP would tell you the truth about your breast cancer status, even if there is bad news? treatment options? | **Ο** | **Ο** | **Ο** | **Ο** | **Ο** | **Ο** |
| 1. I think my HCP cares about breast health as much as I do about my breast health. | **Ο** | **Ο** | **Ο** | **Ο** | **Ο** | **Ο** |
| 1. In the last 12 months, how often did you feel this HCP cared about you as a person? | **Ο** | **Ο** | **Ο** | **Ο** | **Ο** | **Ο** |

1. Using any number from 0 to 10, where 0 means that you do not trust this doctor at all and 10 means that you trust this doctor completely, what number would you use to rate how much you trust this doctor?

| Do not trust this doctor completely | **0** | **1** | **2** | **3** | **4** | **5** | **6** | **7** | **8** | **9** | **10** | Trust this doctor completely |
| --- | --- | --- | --- | --- | --- | --- | --- | --- | --- | --- | --- | --- |
|  | **Ο** | **Ο** | **Ο** | **Ο** | **Ο** | **Ο** | **Ο** | **Ο** | **Ο** | **Ο** | **Ο** |  |

**V. LANGUAGE ACCESS**

**Patients perspective of interpreter service**

*(Never, Rarely, Sometimes, Usually, Occasionally, Always)*

|  | Never | Rarely | Sometimes | Usually | Occasionally | Always |
| --- | --- | --- | --- | --- | --- | --- |
| 1. I often feel I have been treated unfairly at the HCP's office because I do not speak Twi very well. | **Ο** | **Ο** | **Ο** | **Ο** | **Ο** | **Ο** |

1. An interpreter is someone who helps you talk with others who do not speak your language. Interpreters can include friends or family members, staff from the provider's office, or telephone interpreters. During your visits for screening how often did your provider use the services of a licensed interpreter to help you talk with your HCP?

**Ο** Not Available Ο Never, was not necessary as I speak and understand Twi **Ο** Rarely **Ο** Sometimes

**Ο** Usually **Ο** Occasionally **Ο** Always

1. When an interpreter was used at the provider's office, who was the interpreter you used most often when you talked with your HCP?

**Ο** Never used the service of an interpreter as I speak and understand Twi

**Ο** A nurse, clerk, or receptionist from this provider's office

**Ο** A professional interpreter hired by this provider's office to help patients talk with the doctor

**Ο** A bilingual relative acting as an interpreter Other:

1. How often have friends or family members been used by providers as interpreters during your screening at your HCP’s office?

**Ο** Never as I speak and understand Twi **Ο** Rarely **Ο** Sometimes **Ο** Usually **Ο** Occasionally **Ο** Always

**Ο** Never as I have always used the services of a professional interpreter

1. Has there ever been a time in your HCP’s office when you were denied service because of the lack of friends or family members to act as interpreters?

**Ο** Never as I have always used the services of a professional interpreter

**Ο** Never as I speak and understand Twi **Ο** Rarely **Ο** Sometimes **Ο** Usually **Ο** Occasionally **Ο** Always

1. During your visits for screening, did you use friends or family members as interpreters because that was your personal preference?

**Ο** Never as I speak and understand Twi **Ο** Rarely **Ο** Sometimes **Ο** Usually **Ο** Occasionally **Ο** Always

**Ο** Never as I have always used the services of a professional interpreter

1. Has your screening been delayed because you had to wait for an interpreter? Do not include friends or family members.

**Ο** Never as I speak and understand Twi **Ο** Rarely **Ο** Sometimes **Ο** Usually **Ο** Occasionally **Ο** Always

1. During your visits for screening, how often did the interpreter you had most often used at this HCP's office treat you with courtesy and respect? (Do not include friends or family members).

**Ο** I speak and understand Twi, thus never used the services of an interpreter **Ο** Rarely **Ο** Sometimes

**Ο** Usually **Ο** Occasionally **Ο** Always

1. Using any number from 0 to 10 where 0 is the worst interpreter possible and 10 is the best interpreter possible, what number would you use to rate the interpreter you had most often used in the last 12 months? Do not include friends or family members.

| **0** | **1** | **2** | **3** | **4** | **5** | **6** | **7** | **8** | **9** | **10** |
| --- | --- | --- | --- | --- | --- | --- | --- | --- | --- | --- |
| **Ο** | **Ο** | **Ο** | **Ο** | **Ο** | **Ο** | **Ο** | **Ο** | **Ο** | **Ο** | **Ο** |

**Ο** Never used the services of an interpreter as I speak and understand Twi and English

|  | Very well | Well | Not well | Not at all |
| --- | --- | --- | --- | --- |
| 1. How well do you understand English? |  |  |  |  |
| 1. How well do you speak Twi? |  |  |  |  |

**END OF SURVEY**

**Thank you for your time and responses.**
