# Supplementary material for: Factors influencing clinical breast cancer screening: A cross-sectional study among Islamic women in Kumasi Metropolis of Ghana
Source: PLoS One. 2025 May 23;20(5):e0320726. doi: 10.1371/journal.pone.0320726 (PMC12101858; doi:10.1371/journal.pone.0320726)
Supplement: S1 Appendix — (DOCX) [file pone.0320726.s003.docx]

**APPENDIX A**

**Table A1: Association Between Breast Cancer Screening and the Constructs of TCSB**

|  | |  | Breast cancer screening | |  |
| --- | --- | --- | --- | --- | --- |
|  | | **Total** | **Yes** | **No** |  |
| Variables | | **N (%)** | **n (%)** | **n (%)** | **p-value** |
| Perceived Benefits | |  |  |  | **<0.001** |
| Low | 237 (47.4) | 52 (10.4) | 185 (37.0) |  |  |
| High | 263 (52.6) | 96 (19.2) | 167 (33.4) |  |  |
| Knowledge | |  |  |  | **0.014** |
| Low | 252 (50.4) | 62 (12.4) | 190 (38.0) |  |  |
| High | 248 (49.6) | 86 (17.2) | 162 (32.4) |  |  |
| Habits | |  |  |  |  |
| Poor | 250 (50.0) | 42 (8.4) | 208 (41.6) | **<0.001** |  |
| Good | 250 (50.0) | 106 (21.2) | 144 (28.8) |  |  |
| Norms | |  |  |  | **<0.001** |
| Low | 246 (49.2) | 102 (20.4) | 144 (28.8) |  |  |
| High | 254 (50.8) | 46 (9.2) | 208 (41.6) |  |  |
| Facilitating Factors | |  |  |  | **0.012** |
| Poor | 244 (48.8) | 85 (17.0) | 159 (31.8) |  |  |
| Good | 256 (51.2) | 63 (12.6) | 193 (38.6) |  |  |
| Affect | |  |  |  | 0.102 |
| Low | 232 (46.4) | 77 (15.4) | 155 (31.0) |  |  |
| High | 268 (53.6) | 71 (14.2) | 197 (39.4) |  |  |
|  | |  |  |  |  |

p-value= chi-square test with significance level (α=0.05)
